# Bias and precision in true‐score estimation

**DOI:** 10.1111/bmsp.70058

**Published:** 2026-06-15

**Authors:** L. Andries van der Ark, Wilco H. M. Emons, Jules L. Ellis, Klaas Sijtsma

**Affiliations:** ^1^ Research Institute of Child Development and Education University of Amsterdam Amsterdam The Netherlands; ^2^ Tilburg School of Social and Behavioral Sciences Tilburg University Tilburg The Netherlands; ^3^ Faculty of Psychology Open Universiteit Heerlen The Netherlands

**Keywords:** classical test theory, estimation bias, reliability, standard errors, true‐score estimation

## Abstract

We discuss two approaches to estimating the true score from classical test theory, each with a corresponding measure of uncertainty due to measurement error: the classical method with the standard error of measurement (SEM) and Kelley's method with the standard error of estimation (SEE). For both approaches, we examined the bias and sampling variability of the true‐score, SEM and SEE estimators, as these properties were largely unknown. For each estimator, we derived analytic expressions for the bias and proposed approximations for practical bias assessment by omitting cumbersome terms. We also proposed a standard error estimator for each estimator. Simulations were used to assess the accuracy of the bias approximations and standard error estimators and to examine the impact of bias and sampling variability on both true‐score estimation methods. Results indicate that both the bias approximations and standard error estimators are sufficiently accurate. Whereas the classical true‐score estimator is unbiased, Kelley's estimator may exhibit substantial bias for extreme true scores. The bias of the estimated SEM and SEE may also be substantial when reliability is high and the reliability estimator is negatively biased. The impact of sampling variability is typically small.

## 1 INTRODUCTION

Classical test theory (CTT, e.g. Gulliksen, 1950; Lord & Novick, 1968) is the most frequently used framework for estimating test‐score reliability. CTT assumes that any observed test score can be decomposed into a true score and a random measurement error. Ideally, the error‐free true scores rather than error‐laden test scores should be used for measurement. CTT provides two methods to estimate true scores. Both methods provide a point estimate and an uncertainty measure. The *classical method* (e.g. Lord & Novick, 1968, section 7.2) takes the person's observed test score as the estimate of the true score, and the *standard error of measurement* (SEM) as a measure of uncertainty. *Kelley's method* (Kelley, 1947) uses linear regression to estimate the true score as a mixture of a person's observed test score and the mean test score and uses the *standard error of estimate* (SEE) as an uncertainty measure.

The psychometric literature does not unequivocally recommend one method for practical use, and the discussion has been going on for along time (e.g. Campbell & Kenny, 1999, p. 27; Charter & Feldt, 2001; Dudek, 1979; Gulliksen, 1950; Kelley, 1947, p. 411; Patterson, 1955; Stanley & Spence, 2024). As a linear regression estimator of the true score, the Kelley estimator provides the most accurate linear prediction of the true score on average (Holland & Hoskens, 2003). However, it does not improve accuracy uniformly across individuals. Assuming homoscedastic measurement errors, it can be shown that for true scores near the mean, the Kelley estimator is typically more accurate than the classical estimator, whereas for more extreme true scores the reverse is true. Whether the gain in overall accuracy outweighs the local reduction of accuracy is a question that does not admit a general answer, as it may depend on the location of the cut‐off and the context (e.g. Maassen, 2000).

The two measures of uncertainty are also fundamentally different. When homoscedasticity is assumed, the SEM is the estimated standard deviation of the test‐score distribution for a group of individuals with the same true score. When true scores depend linearly on the test scores, the SEE is the estimated standard deviation of the true‐score distribution for a group of individuals with the same test score (e.g. Stanley & Spence, 2024). The debate between the two true‐score estimation methods is conducted at a theoretical level, under the assumption that all relevant parameters are known. In practice, however, true scores and their accompanying uncertainty measures are estimated from finite samples where estimation‐induced bias and sampling variability may obscure the theoretical properties. In this study, we examine these issues.

This study has two goals. First, we derive analytical expressions for the bias of the true‐score estimators, the SEM and the SEE. The resulting expressions have limited practical use, however, because they involve quantities that are typically unknown prior to data collection and difficult to specify a priori (e.g. sampling covariances between estimators and higher‐order aspects of sampling variability). To obtain practically useful approximations, we therefore consider reduced expressions—referred to as *bias approximations*—in which such terms are omitted, as they are expected to have limited impact on the bias. These bias approximations depend primarily on quantities, such as reliability and its bias, for which researchers often have substantive expectations, and therefore have greater practical applicability. We evaluate their accuracy in a simulation study. Second, we provide standard error estimators for the true‐score, SEM and SEE estimators, to assess the impact of sampling variability on true‐score estimates and their associated measures of uncertainty. We assume that test scores are obtained from a single administration of the test to a sample of N persons.

The remainder of this paper is organized as follows. First, we discuss the two true‐score estimation methods. Second, we derive analytical expressions for the bias and the proposed bias approximations. Third, we derive the standard error estimators. Fourth, we examine the accuracy of the bias approximations and standard error estimators in a simulation study. Fifth, we assess the accuracy of the true‐score, SEM and SEE estimators. Sixth, we provide plots giving insight into the magnitude of bias. We conclude with a discussion.

## 2 THEORETICAL FRAMEWORK OF TRUE‐SCORE ESTIMATION

### 2.1 Classical test theory

Let P denote a population of persons, indexed $v\;\left(v=1,2,\ldots \right)$, let R denote a set of replications (i.e. repeated test administrations under identical circumstances), indexed by $k\;\left(k=1,2,\ldots \right)$. Let random variable $X_{v}$ denote the test score of person v at a randomly selected replication. The distribution of $X_{v}$, denoted $f\left(X_{v}\right)$, is called the *propensity distribution* of person v, and $f\left(X_{v}\right)$ need not have a specific shape. Person v's *true score*, denoted by $\tau_{v}$, is operationally defined (i.e. it is a conveniently chosen mathematical definition) as the expected value of $f\left(X_{v}\right)$ value over replications:

(1) $$\tau_{v}\equiv E\left(X_{v}\right),$$

where $E\left(\right)$ denotes the expected value. Deviations from the mean are measurement errors, denoted $E_{v}$; that is,

(2) $$E_{v}=X_{v}-\tau_{v}.$$

Equation 2 is the CTT model. By implication, we find that

(3) $$E\left(E_{v}\right)=E\left(X_{v}-\tau_{v}\right)=0.$$

The variance of the propensity distribution, denoted by $\mathrm{Var}\left(X_{v}\right)$, reflects within‐person variability due to random measurement error. CTT allows that $\mathrm{Var}\left(X_{v}\right)$ varies across persons, and smaller within‐person variability implies greater measurement precision (e.g. Mellenbergh, 1996; Sijtsma et al., 2024).

For test scores obtained from multiple persons in a single test administration, CTT assumes a double stochastic process. First, persons are sampled from P. Then, for each person in the sample, a test score is obtained by a random draw from their propensity distribution. Let the random variable X denote the test score, with $E\left(X\right)=\mu_{X}$ and $\mathrm{Var}\left(X\right)=\sigma_{X}^{2}$. Furthermore, let the random variable denoted by Greek upper‐case tau, T. denote the true score, with $E\left(Τ\right)=\mu_{Τ}$ and $\mathrm{Var}\left(Τ\right)=\sigma_{Τ}^{2}$. Finally, let random variable E denote the random measurement error, with $E\left(E\right)=\mu_{E}$ and $\mathrm{Var}\left(E\right)=\sigma_{E}^{2}$. The CTT model is usually written as

(4) X=T+E.

It follows from Equations 1 and 2 that in the population T and E are uncorrelated. As a result,

(5) $$\sigma_{X}^{2}=\sigma_{T}^{2}+\sigma_{E}^{2}.$$

Because of Equation 3, in the population we have $E\left[E\left(E_{v}\right)\right]=\mu_{E}=0$. Then, it follows from Equations 1 and 2 that $\mu_{X}=\mu_{Τ}.$

The main goal of CTT is to determine the reliability of the test score in the population of interest: Which proportion of the test‐score variance is true‐score variance, or equivalently, which proportion of the test‐score variance is not error variance? Reliability $\rho_{X}$ is defined as

(6) $$\rho_{X}=\frac{\sigma_{T}^{2}}{\sigma_{X}^{2}}=1-\frac{\sigma_{E}^{2}}{\sigma_{T}^{2}}$$

(Lord & Novick, 1968, p. 61). Because in Equation 6, both $\sigma_{T}^{2}$ and $\sigma_{E}^{2}$ are unobservable, $\rho_{X}$ cannot be directly estimated from the data. Methods for estimating reliability have been proposed that circumvent this problem and do not require additional assumptions about the test data. These methods provide lower bounds to the reliability (e.g. Sijtsma et al., 2024). Examples include the six methods proposed by Guttman (1945), among which is coefficient $\lambda_{3}$, later called coefficient α by Cronbach (1951), as well as the greatest lower bound (Jackson & Agunwamba, 1977; Woodhouse & Jackson, 1977; Woodward & Bentler, 1978).

### 2.2 The classical method

#### 2.2.1 Estimating the true score

The classical method estimates a person's true score by means of the observed test score. Using C for the classical method, we write the classical estimator as follows:

(7) $${\hat{\tau }}_{v}^{C}=X_{v}.$$

Estimator ${\hat{\tau }}_{v}^{C}$ does not require data from other persons. Assuming all propensity distributions are normally distributed, Mellenbergh (1996) showed that a person's observed test score is the maximum likelihood estimator of the person's true score.

#### 2.2.2 Estimating the SEM

Because of Equation 7, the variance of the true‐score estimate ${\hat{\tau }}_{v}^{C}$ equals the variance of person v's propensity distribution, $\mathrm{Var}\left(X_{v}\right)$, which cannot be estimated from a single test score. However, it can be derived (e.g. Mellenbergh, 1996) that the expected value of all individual error variances equals the measurement‐error variance; that is,

(8) $$E\left[\mathrm{Var}\left(X_{v}\right)\right]=\sigma_{E}^{2}.$$

In practical applications, $\sigma_{E}^{2}$ typically serves as an approximation of $\mathrm{Var}\left(X_{v}\right)$ for all persons. Standard deviation $\sigma_{E}$ is the SEM, which is derived from the right‐hand side of Equation 6 as

(9) $$\sigma_{E}=\sigma_{X}\sqrt{1-\rho_{X}}.$$

Assuming homoscedasticity, the SEM can be considered the estimated standard deviation of the distribution of X for a group of persons with the same test score Τ (e.g. Stanley & Spence, 2024), and the SEM can be used to construct Wald confidence intervals for a person's true score.

In samples, the SEM is estimated by

(10) $${\hat{\sigma }}_{E}={\hat{\sigma }}_{X}\sqrt{1-{\hat{\rho }}_{X}},$$

where ${\hat{\sigma }}_{X}$ denotes the sample standard deviation of test score X, and ${\hat{\rho }}_{X}$ represents an estimate of the reliability. It may be noted that CTT does not assume an equal SEM for all persons, but test practitioners use the SEM for convenience. However, under additional assumptions, SEM methods conditional on the test score have been proposed (e.g. Emons, 2023; Pfadt et al., 2026).

### 2.3 Kelley's method

#### 2.3.1 Estimating the true score

Kelley's method uses the linear regression of the true score on the test score to obtain a predicted true score (Kelley, 1947, p. 409; also, see Lord & Novick, 1968, p. 65). In the population, where $\rho_{X}$ and $\mu_{X}$ are known, the predicted true score is given by

(11) $${\tilde{\tau }}_{v}^{K}=\rho_{X}X_{v}+\left(1-\rho_{X}\right)\mu_{X},$$

where $\left(1-\rho_{X}\right)\mu_{X}$ is the intercept, $\rho_{X}$ is the regression coefficient, and ${\tilde{\tau }}_{v}^{K}$ is the best linear predictor (BLP) of true score $\tau_{v}$ (e.g. Holland & Hoskens, 2003). If the regression of Τ on X is linear—for example, if $\left(Τ,E\right)$ follows a bivariate normal distribution—then ${\tilde{\tau }}_{v}^{K}=E\left(Τ|X\right)$. Equation 11 shows that, as reliability $\rho_{X}$ increases, the person's test score receives greater weight, and the group mean receives less weight in the estimation of the true score. In samples, the Kelley estimator, denoted ${\hat{\tau }}_{v}^{K}$ is obtained by replacing $\rho_{X}$ and $\mu_{X}$ by sample estimates,

(12) $${\hat{\tau }}_{v}^{K}={\hat{\rho }}_{X}X_{v}+\left(1-{\hat{\rho }}_{X}\right)\;{\hat{\mu }}_{X}.$$

There are two notable differences between the classical and Kelley's true‐score estimator. First, the Kelley true‐score estimator for person v (Equation 12) is affected by ${\hat{\rho }}_{X}$ and ${\hat{\mu }}_{X}$. Therefore, two persons who have the same test scores on the same test may have different true‐score estimates if ${\hat{\rho }}_{X}$ and ${\hat{\mu }}_{X}$ were estimated from different norm samples. The classical method always produces the same estimate (Equation 7). Second, for the classical method, the expected individual true‐score estimate equals $\tau_{v}$ (Equation 1), whereas it follows from Equations 1 and 11 that for Kelley's method, the expected individual true‐score estimate equals

(13) $$\tau_{v}^{*}=E\left({\tilde{\tau }}_{v}^{K}\right)=\rho_{X}\;E\left(X_{v}\right)+\left(1-\rho_{X}\right)\mu_{X}=\rho_{X}\tau_{v}+\left(1-\rho_{X}\right)\mu_{X}.$$

Equation 13 shows that $\tau_{v}^{*}$ is a linear transformation of $\tau_{v}$, and the two are therefore perfectly correlated. Equation 13 also shows that generally $E\left({\tilde{\tau }}_{v}^{K}\right)\neq \tau_{v}$. Therefore, even if ${\hat{\rho }}_{X}$ and ${\hat{\mu }}_{X}$ in Equation 12 are unbiased estimators, ${\hat{\tau }}_{v}^{K}$ is a biased estimator of $\tau_{v}$ because, in general, $E\left({\tilde{\tau }}_{v}^{K}\right)\neq \tau_{v}$. This bias comes at the benefit of more precise estimates by reducing the overall accuracy.

#### 2.3.2 Estimating the SEE

Following Lord and Novick (1968, p. 67), let $\epsilon =\tau_{v}^{*}-\tau_{v}$, then the standard error of estimation (SEE) equals

(14) $$\sigma_{\epsilon }=\sigma_{T}\sqrt{1-\rho_{X}}.$$

Substituting $\sigma_{T}=\sqrt{\rho_{X}}$ $\sigma_{X}$ (cf. Equation 6), results in

(15) $$\sigma_{\epsilon }=\sigma_{X}\sqrt{\rho_{X}}\sqrt{1-\rho_{X}}$$

(Lord & Novick, 1968, p. 67). In samples, Equation 15 is estimated using

(16) $${\hat{\sigma }}_{\epsilon }={\hat{\sigma }}_{X}\sqrt{{\hat{\rho }}_{X}}\;\sqrt{1-{\hat{\rho }}_{X}}.$$

When the regression of Τ on X is linear, and assuming homoscedasticity, the SEE can be considered the estimated standard deviation of the distribution of T for a group of persons with the same test score X, and the SEE can be used to construct a *credible interval* around X. Hence, the SEE and the SEM are conceptually different measures, and the width of a 95% confidence interval obtained using the SEM and the width of a 95% credible interval obtained using the SEE cannot be directly compared (e.g. see Morey et al., 2016 for a thorough discussion of interval estimators; also, see Stanley & Spence, 2024).

## 3 BIAS AND BIAS APPROXIMATIONS

For a generic parameter λ, bias is defined as $\text{Bias}\left(\hat{\lambda }\right)=E\left(\hat{\lambda }\right)-\lambda$, where the expectation is taken across different samples. In this section, we discuss the bias of the classical true‐score estimator (Equation 7), Kelley's true‐score estimator (Equation 12), the SEM estimator (Equation 10) and the SEE estimator (Equation 16).

### 3.1 Bias of the true‐score estimators

For the classical method, ${\hat{\tau }}_{v}^{C}$ is an unbiased estimator of $\tau_{v}$ (Appendix A; also, see Lord & Novick, 1968, section 7.2). For Kelley's method, bias is defined as $\text{Bias}\left({\hat{\tau }}_{v}^{K}\right)=E\left({\hat{\tau }}_{v}^{K}\right)-\tau_{v}$, which can be decomposed into

(17) $$\text{Bias}\left({\hat{\tau }}_{v}^{K}\right)=\left[E\left({\hat{\tau }}_{v}^{K}\right)-\tau_{v}^{*}\right]+\left[\tau_{v}^{*}-\tau_{v}\right],$$

where $E\left({\hat{\tau }}_{v}^{K}\right)-\tau_{v}^{*}$ is *finite‐sample bias*, and $\tau_{v}^{*}-\tau_{v}$ is *asymptotic bias*, the systematic difference between the true score and Kelley's expected true‐score estimate (Equation 13) due to the linear transformation of the test scores. The finite‐sample bias equals

(18) $$E\left({\hat{\tau }}_{v}^{K}\right)-\tau_{v}^{*}=\text{Bias}\left({\hat{\rho }}_{X}\right)\left[\tau_{v}-\mu_{X}\right]+\mathrm{Cov}\left({\hat{\rho }}_{X},X_{v}\right)+\mathrm{Cov}\left({\hat{\rho }}_{X},{\hat{\mu }}_{X}\right)$$

(Appendix B), where $\mathrm{Cov}\left(\right)$ denotes the covariance. The total bias (Equation 17) then becomes

(19) $$\text{Bias}\left({\hat{\tau }}_{v}^{K}\right)=\text{Bias}\left({\hat{\rho }}_{X}\right)\left[\tau_{v}-\mu_{X}\right]+\mathrm{Cov}\left({\hat{\rho }}_{X},X_{v}\right)+\mathrm{Cov}\left({\hat{\rho }}_{X},{\hat{\mu }}_{X}\right)+\left[\tau_{v}^{*}-\tau_{v}\right].$$

Appendix B provides conditions for which $\mathrm{Cov}\left({\hat{\rho }}_{X},X_{v}\right)=0$ and $\mathrm{Cov}\left({\hat{\rho }}_{X},{\hat{\mu }}_{X}\right)=0.$

For bias approximation, we omitted the sampling covariances between estimators—$\mathrm{Cov}\left({\hat{\rho }}_{X},X_{v}\right)$ and $\mathrm{Cov}\left({\hat{\rho }}_{X},{\hat{\mu }}_{X}\right)$—in Equation 19. Let $\tilde{\text{Bias}}\left({\hat{\tau }}_{v}^{K}\right)$ denoted the bias approximation of ${\hat{\tau }}_{v}^{K}$, then

(20) $$\tilde{\text{Bias}}\left({\hat{\tau }}_{v}^{K}\right)=\text{Bias}\left({\hat{\rho }}_{X}\right)\left[\tau_{v}-\mu_{X}\right]+\left[\tau_{v}^{*}-\tau_{v}\right].$$

Based on the conditions in Appendix B, we expect that the magnitude of the sampling covariances is small compared with the other terms in Equation 19. Below, using simulations, we examined the accuracy of Equation 20 as a bias approximation.

### 3.2 Bias of the estimated SEM and SEE

Appendix C shows that the bias of the estimated squared SEM equals

(21) $$\text{Bias}\left({\hat{\sigma }}_{E}^{2}\right)=\sigma_{X}^{2}\left[-\text{Bias}\left({\hat{\rho }}_{X}\right)\right]+\mathrm{Cov}\left({\hat{\sigma }}_{X}^{2},{\hat{\rho }}_{X}\right).$$

Using a second‐order Taylor expansion of Equation 19, the bias of the estimated SEM can be approximated by:

(22) $$\hat{\text{Bias}}\left({\hat{\sigma }}_{E}\right)=\frac{1}{2\sigma_{X}\sqrt{1-\rho_{X}}}\left\{-\sigma_{X}^{2}\left[\text{Bias}\left({\hat{\rho }}_{X}\right)\right]+\mathrm{Cov}\left({\hat{\sigma }}_{X}^{2},{\hat{\rho }}_{X}\right)\right\}-\frac{1}{8\sigma_{E}^{3}}\mathrm{Var}\left({\hat{\sigma }}_{E}^{2}\right)$$

(see Appendix C). To approximate the bias of the SEM estimator in practice, we omitted $\mathrm{Cov}\left({\hat{\sigma }}_{X}^{2},{\hat{\rho }}_{X}\right)$ and $\frac{1}{8\sigma_{E}^{3}}\mathrm{Var}\left({\hat{\sigma }}_{E}^{2}\right)$ from Equation 22. Let $\tilde{\text{Bias}}\left({\hat{\sigma }}_{E}\right)$ denoted the bias approximation of ${\hat{\sigma }}_{E}$, then

(23) $$\tilde{\text{Bias}}\;\left({\hat{\sigma }}_{E}\right)=\frac{1}{2\sigma_{X}\sqrt{1-\rho_{X}}}\left\{-\sigma_{X}^{2}\left[\text{Bias}\left({\hat{\rho }}_{X}\right)\right]\right\}=-\frac{\sigma_{X}}{2\sqrt{1-\rho_{X}}}\;\text{Bias}\left({\hat{\rho }}_{X}\right).$$

Appendix C discusses a condition in which $\mathrm{Cov}\left({\hat{\sigma }}_{X}^{2},{\hat{\rho }}_{X}\right)$ equals 0, but in general it does not, and exclusions of this term may affect the bias approximation. Because $\mathrm{Var}\left({\hat{\sigma }}_{E}^{2}\right)$ decreases as N increases, the effect of not including this term in the bias approximation (Equation 23) is expected to be small for large N.

Appendix D shows that the bias of the estimated squared SEE equals

(24) $$\text{Bias}\left({\hat{\sigma }}_{\epsilon }^{2}\right)=\sigma_{X}^{2}\left[\left(1-2\rho_{X}\right)\text{Bias}\left({\hat{\rho }}_{X}\right)-\text{Bias}{\left({\hat{\rho }}_{X}\right)}^{2}-\mathrm{Var}\left({\hat{\rho }}_{X}\right)\right]+\mathrm{Cov}\left({\hat{\sigma }}_{X}^{2},{\hat{\rho }}_{X}-{\hat{\rho }}_{X}^{2}\right).$$

Using a second‐order Taylor expansion, Appendix D shows that the bias of the estimated SEE can be approximated by

(25) $$\hat{\text{Bias}}\left({\hat{\sigma }}_{\epsilon }\right)=\frac{\sigma_{X}\left[-\text{Bias}{\left({\hat{\rho }}_{X}\right)}^{2}+\left(1-2\rho_{X}\right)\text{Bias}\left({\hat{\rho }}_{X}\right)-\mathrm{Var}\left({\hat{\rho }}_{X}\right)\right]+\mathrm{Cov}\left({\hat{\sigma }}_{X}^{2},{\hat{\rho }}_{X}-{\hat{\rho }}_{X}^{2}\right)}{2\sqrt{\rho_{X}\left(1-\rho_{X}\right)}}-\frac{1}{8\sigma_{\epsilon }^{3}}\mathrm{Var}\left({\hat{\sigma }}_{\epsilon }^{2}\right),$$

a quadratic function of $\text{Bias}\left({\hat{\rho }}_{X}\right)$. To approximate the bias of the SEE estimator in practice, we omitted $\mathrm{Cov}\left({\hat{\sigma }}_{X}^{2},{\hat{\rho }}_{X}-{\hat{\rho }}_{X}^{2}\right)$, $\frac{1}{8\sigma_{\epsilon }^{3}}\mathrm{Var}\left({\hat{\sigma }}_{\epsilon }^{2}\right)$ and $\mathrm{Var}\left({\hat{\rho }}_{X}\right)$ from Equation 25. Let $\tilde{\text{Bias}}\left({\hat{\sigma }}_{\epsilon }\right)$ denote the bias approximation of ${\hat{\sigma }}_{\epsilon }$, then

(26) $$\tilde{\text{Bias}}\left({\hat{\sigma }}_{\epsilon }\right)=\frac{\sigma_{X}\left[-\text{Bias}{\left({\hat{\rho }}_{X}\right)}^{2}+\left(1-2\rho_{X}\right)\text{Bias}\left({\hat{\rho }}_{X}\right)\right]}{2\sqrt{\rho_{X}\left(1-\rho_{X}\right)}}.$$

The treatment of the omitted terms is similar to that used to obtain the bias approximation of the SEM estimator (Equation 23). In Equation 25, $\mathrm{Cov}\left({\hat{\sigma }}_{X}^{2},{\hat{\rho }}_{X}-{\hat{\rho }}_{X}^{2}\right)=0$, if ${\hat{\sigma }}_{X}^{2}$ and ${\hat{\rho }}_{X}$ are obtained from different samples, but in general this term does not equal zero, and its exclusions may affect the bias approximation. Both $\mathrm{Var}\left({\hat{\sigma }}_{E}^{2}\right)$ and $\mathrm{Var}\left({\hat{\rho }}_{X}\right)$ decrease as N increases. Therefore, their exclusion in the bias approximation is expected to have only minor effects for large N. Below, using simulations, we examined the accuracy of Equations 23 and 26 as a bias approximation.

## 4 STANDARD ERRORS

For a generic parameter λ, the extent of sampling variability can be expressed by the sampling variance, $\mathrm{Var}\left(\hat{\lambda }\right)$ or standard error $\mathrm{SE}\left(\hat{\lambda }\right)=\sqrt{\mathrm{Var}\left(\hat{\lambda }\right)}$. *Precision* is defined as $1/\mathrm{Var}\left(\hat{\lambda }\right)$. As the classical true‐score estimator (Equation 7) is affected only by measurement error, and not by sampling variability, its standard error is zero by definition. In the remainder of this section, we derive analytic standard errors for Kelley's true‐score estimates (Equation 12), the SEM estimator (Equation 10) and the SEE estimator (Equation 16) under the multinomial sampling scheme.

Three comments are in order. First, persons having the same test score also receive the same estimated true score, ${\hat{\tau }}_{v}^{K}$. Therefore, the number of estimated true scores equals the number of observed test scores in the data. Assume that L≤N test scores have been observed in the data, denoted $X_{\left(1\right)},\ldots ,X_{\left(L\right)}$. Then ${\hat{\tau }}^{K}={\left[{\hat{\tau }}^{K}\left|X_{\left(1\right)},{\hat{\tau }}^{K}\right|X_{\left(2\right)},\ldots ,{\hat{\tau }}^{K}|X_{\left(L\right)}\right]}^{\prime }$ is the vector containing the L true‐score estimates in the sample, where the apostrophe denotes the transposition. Collecting these L true‐score estimates in a vector is convenient as it allows simultaneous estimation of the standard errors for all true‐score estimates in the sample.

Second, for deriving the standard errors we used a two‐step procedure involving recursive computation, which has been described in detail elsewhere (e.g. Koopman et al., 2020; Kuijpers et al., 2013; Oosterhuis et al., 2017; Van der Ark, 2025). Therefore, we have diverted the full explanation of the two‐step method to the Data S1 (Section 1) and describe the procedure only briefly here.

Third, ${\hat{\tau }}^{K}$, ${\hat{\sigma }}_{E}$ and ${\hat{\sigma }}_{\epsilon }$ depend on the particular reliability estimator. Therefore, their standard errors depend this estimator as well. To keep the task manageable, we derived these standard errors only for the case in which test‐score reliability is estimated using Cronbach's alpha. However, they can also be derived for other reliability estimators with known standard errors.

In the first step of the two‐step procedure, the test data are written as a multiway contingency table that contains the frequencies of all possible item‐score patterns. The cells with frequencies that pertain to observed item‐score patterns are stacked in a vector n. Let $D_{n}$ denote a diagonal matrix with vector n on the diagonal, let 1 denote the unity vector, and let $Y^{-1}$ denote the inverse of matrix Y. Assuming that n follows a multinomial distribution, the estimated covariance matrix of n, denoted ${\hat{V}}_{n}$, equals (e.g. Agresti, 2002)

(27) $${\hat{V}}_{n}=D_{n}-{\mathrm{nn}}^{\prime }{\left(1^{\prime }n\right)}^{-1}.$$

For parameter vector estimate $\hat{\lambda }$, a recursive method is used to derive $G_{\hat{\lambda }}$, the vector of first‐order partial derivatives (Jacobian) of $\hat{\lambda }$ with respect to n. If there is only a single parameter estimate, as is the case for ${\hat{\sigma }}_{E}$, and ${\hat{\sigma }}_{\epsilon }$, $\hat{\lambda }$ is a scalar. In the second step, the delta method, based on a first‐order Taylor approximation, is used to derive $\mathrm{Var}\left(\hat{\lambda }\right)$: $\hat{\mathrm{Var}}\left(\hat{\lambda }\right)={G_{\hat{\lambda }}}^{\prime }{\hat{V}}_{n}G_{\hat{\lambda }}$, and the estimated standard error equals the square root of $\hat{\mathrm{Var}}\left(\hat{\lambda }\right)$.

Suppose we have obtained n and ${\hat{V}}_{n}$. Then, consider design matrices $A_{1}$, $A_{2}$, $A_{3}$, $A_{4}$, $A_{5}$, $A_{5}^{*}$, $A_{5}^{†}$, $A_{6}$, $A_{6}^{*}$, $A_{6}^{†}$, $A_{7}$ and $A_{8}$ in Table E1 of Appendix E, and consider the corresponding functions $g_{i}$ $g_{i}^{*}$, and $g_{i}^{†}$, and $G_{i}$ $G_{i}^{*}$, and $G_{i}^{†}$ in Table E2 in Appendix E. After some tedious algebra, it can be derived that when coefficient alpha is used as a reliability estimator, then ${\hat{\tau }}^{K}=g_{{\hat{\tau }}^{K}}=g_{8}$, ${\hat{\sigma }}_{E}=g_{{\hat{\sigma }}_{E}}=g_{6}^{*}$ and ${\hat{\sigma }}_{\epsilon }=g_{{\hat{\sigma }}_{\epsilon }}=g_{6}^{†}$; and that the corresponding Jacobians are $\frac{\partial {\hat{\tau }}^{K}}{\partial n^{\prime }\;}=G_{{\hat{\tau }}^{K}}=G_{8}$, $\frac{\partial {\hat{\sigma }}_{E}}{\partial n^{\prime }\;}=G_{{\hat{\sigma }}_{E}}=G_{6}^{*}$ and $\frac{{\hat{\sigma }}_{\epsilon }}{\partial n^{\prime }\;}=G_{{\hat{\sigma }}_{\epsilon }}=G_{6}^{†}$. Let $\text{Diag}\left(Y\right)$ denote the diagonal elements of a square matrix Y. Using the estimate of $V_{n}$ in Equation 27, and using the delta method based on a first‐order Taylor approximation, the standard errors of ${\hat{\tau }}^{K}$, ${\hat{\sigma }}_{E}$ and ${\hat{\sigma }}_{\epsilon }$ are estimated by

(28) $$\hat{\mathrm{SE}}\left({\hat{\tau }}^{K}\right)=\sqrt{\text{Diag}\left(G_{{\hat{T}}^{K}}^{\prime }{\hat{V}}_{n}G_{{\hat{T}}^{K}}\right)},$$

(29) $$\hat{\mathrm{SE}}\left({\hat{\sigma }}_{E}\right)=\sqrt{G_{{\hat{\sigma }}_{E}}^{\prime }{\hat{V}}_{n}G_{{\hat{\sigma }}_{E}}},$$

and

(30) $$\hat{\mathrm{SE}}\left({\hat{\sigma }}_{\epsilon }\right)=\sqrt{G_{{\hat{\sigma }}_{\epsilon }}^{\prime }{\hat{V}}_{n}G_{{\hat{\sigma }}_{\epsilon }}},$$

respectively. The Data S1 (Section 1.3) provide R functions for the standard error estimates in Equations 28, 29 and 30.

## 5 VALIDITY OF THE BIAS APPROXIMATIONS AND STANDARD ERRORS

Under several conditions, we compared the bias expressions (Equations 19, 22 and 25), the bias approximations (Equations 20, 23 and 26) and the bias obtained from simulated data, which we refer to as the empirical bias. If the bias approximations are close to both the bias expressions and the empirical bias, this supports the idea that these approximations can be used to examine bias in practice. We also compared the estimated standard errors (Equations 28, 29 and 30) with empirical standard errors obtained from replicated simulated data to assess their accuracy.

### 5.1 Method

#### 5.1.1 Data‐generating model

A two‐parameter logistic item‐response theory (IRT; Birnbaum, 1968) model for J dichotomous items served as the data‐generating model. We selected item‐parameters sets that approximately produced prespecified values for both the reliability ($\rho_{X}$) and the bias of the reliability estimator (see, Data S1, Section 2). The difference between the prespecified and produced reliability values was < .00001. The discrete latent variable Θ, whose realizations are denoted by θ, took 10,001 equally spaced values between −5 and 5 with a step size of .001. The distribution of Θ, $P\left(\Theta =\theta \right)$, approximated a standard normal distribution. Cronbach's alpha (α) was used as the reliability estimator.

The following relevant parameters were computed from the data‐generating model: $\tau_{\theta }$ (i.e. the true score for a person with Θ=θ; θ=−5,−4.999,…,4.999,5; $0<\tau_{\theta }<J$), $\sigma_{T}^{2}$, $\mu_{X}$, $\sigma_{X}^{2}$ and α (for details, see Appendix F). From these parameters, the reliability (Equation 6), the SEM (Equation 9), the SEE (Equation 15) and the bias of the reliability estimator ($\alpha -\rho_{X}$) were computed. A true standard error was approximated by the empirical standard error: the standard deviation of the estimated statistic over the replications.

#### 5.1.2 Independent variables

We used a fully crossed design with four between‐subject independent variables and one within‐subject independent variable:

- Number of items (J) had two levels (J=6 and J=12). We selected relatively small numbers of items, as we expected that any mismatch between the analytical expressions and simulation results would be largest for small numbers of items.
- Reliability ($\rho_{X}$) had four levels ($\rho_{X}=.6$, .7, .8 and .9), values that are often used as benchmarks (e.g. Nunnally, 1978).
- Bias of the reliability estimator, Bias $\left({\hat{\rho }}_{X}\right)$, had four levels (Bias $\left({\hat{\rho }}_{X}\right)=0,-.02,-.05$ and −.1), representing no bias, small bias, large bias and excessive bias, respectively.
- Sample size (N) had three levels (N=200,500,1000), representing small, medium and large samples, respectively.
- Parameter estimators of interest, a within‐subject variable, had five levels: estimators for a low true score, an average true score, a high true score, the SEM and the SEE. Let $\tau \left(\theta \right)$ denote the true score for Θ=θ. To examine bias, $\tau \left(-2\right)$, $\tau \left(0\right)$ and $\tau \left(2\right)$ represented the low, average and high true score, respectively. Consequently, each sample consisted of three fixed latent‐variable values (θ=−2,0,2) and N−3 independently drawn latent‐variable values. Standard errors of true‐score estimates were examined at three fixed test scores: $x=\frac{1}{6}J,\frac{1}{2}J,$ and $\frac{5}{6}J$.

#### 5.1.3 Dependent variables

First, we examined the magnitude of the terms that were omitted from the bias expressions. Second, for the parameter estimators of interest ($\hat{\tau }\left(-2\right)$, $\hat{\tau }\left(0\right)$, $\hat{\tau }\left(2\right)$, ${\hat{\sigma }}_{E}$, ${\hat{\sigma }}_{\epsilon }$) we compared the exact bias, the bias approximation and the empirical bias (i.e. bias obtained from the simulation study), by examining their correlations across all 96 conditions, and the differences between the bias and its approximation. Furthermore, for the same parameter estimators, we compared the estimated standard errors with the empirical standard errors by examining their correlations across all 96 conditions, as well as the root mean squared error (RMSE) and coverage rate within each condition.

#### 5.1.4 Procedure

For both bias and standard errors, and for each parameter estimator of interest, we used a fully crossed design with 2×4×4×3=96 conditions. Each condition was replicated 5000 times, resulting in 5000 datasets per condition. The main results are presented in the paper, additional results are provided in the Data S1 (Section 3).

### 5.2 Results and discussion

#### 5.2.1 Bias

For the terms that were omitted from the bias expressions to obtain the bias approximations, Table 1 shows the distribution of their values across all 96 conditions, providing a first glance of the contribution of these terms to the bias, which ranged from −.0104 to .0250. The full tables are available in the Data S1 (Section 3.1). In general, these values appear to be small. $\mathrm{Cov}\left({\hat{\sigma }}_{X}^{2},{\hat{\rho }}_{X}\right)$ and $\mathrm{Cov}\left({\hat{\sigma }}_{X}^{2},{\hat{\rho }}_{X}-{\hat{\rho }}_{X}^{2}\right)$ have the largest impact; omitting them leads to an overestimation in the bias of the estimated SEM and an underestimation in the bias of the estimated SEE, respectively. The effects of these terms on the bias is discussed next.

**TABLE 1:** Summary statistics of the distributions of the terms omitted to simplify the bias expressions, across the 96 study conditions.

Term	Equation	Min	Q1	Median	Mean	Q3	Max
$\mathrm{Cov}\left({\hat{\rho }}_{X},X_{l}\right)$	19	−104	−12	−3	−10	−1	2
$\mathrm{Cov}\left({\hat{\rho }}_{X},X_{m}\right)$	19	−9	−1	0	0	2	13
$\mathrm{Cov}\left({\hat{\rho }}_{X},X_{h}\right)$	19	0	1	5	12	14	98
$\mathrm{Cov}\left({\hat{\rho }}_{X},{\hat{\mu }}_{X}\right)$	19	−17	0	0	1	2	35
$\mathrm{Cov}\left({\hat{\sigma }}_{X}^{2},{\hat{\rho }}_{X}\right)$	22	3	13	34	1	75	250
$1/8\sigma_{E}^{3}\;\mathrm{Var}\left({\hat{\sigma }}_{E}^{2}\right)$	22	0	0	1	2	2	20
$\mathrm{Cov}\left({\hat{\sigma }}_{X}^{2},{\hat{\rho }}_{X}-{\hat{\rho }}_{X}^{2}\right)$	25	−90	−26	−10	−18	−4	3
$\mathrm{Var}\left({\hat{\rho }}_{X}\right)$	25	0	1	3	6	7	42
$1/8\sigma_{\epsilon }^{3}\;\mathrm{Var}\left({\hat{\sigma }}_{\epsilon }^{2}\right)$	25	0	0	1	3	3	28

*Note*: All values were multiplied by 10,000 to improve readability.

For Kelley's true‐score estimators and the SEE estimator, the bias, the bias approximations and the empirical bias correlated almost perfectly (Table 2, first three columns). For the SEM estimator, the correlations were slightly lower (approximately .98), which is mainly due to the conditions with an unbiased reliability estimator (Figure 1). In these conditions, the bias approximation (Equation 23) cannot be negative whereas the actual bias (Equation 22) can be due the term $\mathrm{Cov}\left({\hat{\sigma }}_{X}^{2},{\hat{\rho }}_{X}\right)$, which has a negative impact on the bias. Figures for the other four estimators (see Data S1, Section 3.2) suggest that the differences between bias and its approximation are very small, with all points clustered along the diagonal line.

**TABLE 2:** Correlations across the 96 study conditions between bias, bias approximation and empirically determined bias for the five parameters of interest.

Estimator	Bias			SE
	ExB v. BAp	ExB v. EmB	BAp v. EmB	EsS v. EmS
$\hat{\tau }\left(\theta =-2\right)$	.9997	.9995	.9997	.9995
$\hat{\tau }\left(\theta =0\right)$	.9995	.9932	.9935	.9992
$\hat{\tau }\left(\theta =2\right)$	.9999	.9996	.9997	.9989
${\hat{\sigma }}_{E}$	.9870	.9857	.9970	.9997
${\hat{\sigma }}_{\epsilon }$	.9997	.9980	.9984	.9992

Abbreviations: BAp, Bias approximation; EmB, Empirical bias; EmS, Empirical standard error; EsS, Estimated standard error; ExB, Exact bias (in case of SEM and SEE, the bias is in fact an approximation).

**FIGURE 1:**
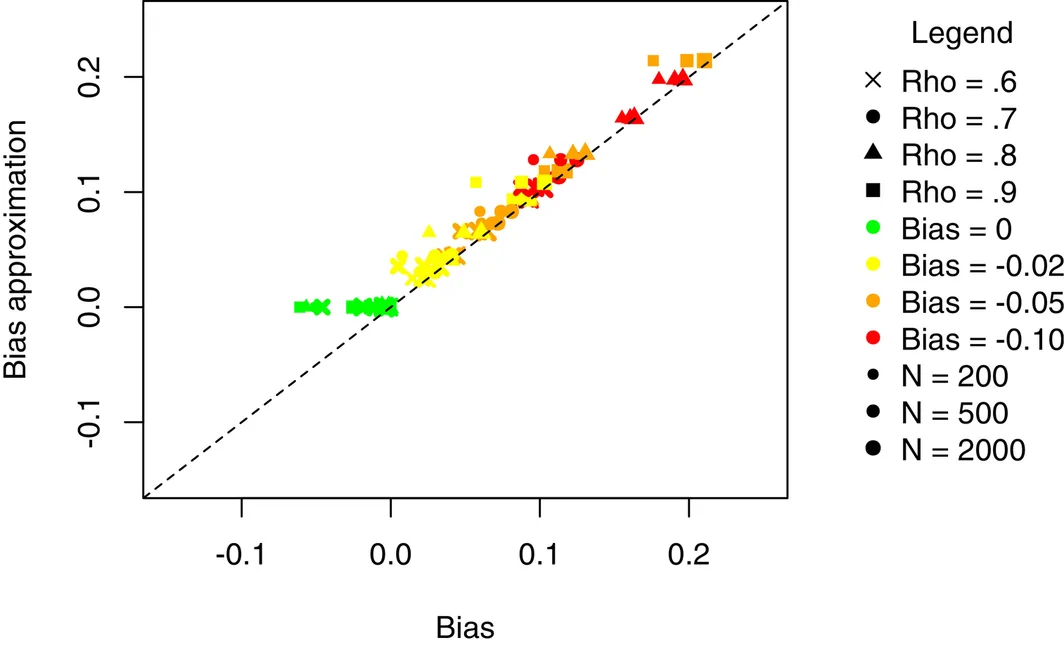
Bias and bias approximation of the estimated standard error of measurement (SEM) for the 96 conditions in the simulation study.

#### 5.2.2 Standard errors

The correlation between the estimated and empirical standard errors was close to .999 (Table 2, Column 4). Table 3 shows a summary of the distribution of the RMSEs of the standard error estimates across all 96 conditions (for full tables, see Data S1, Section 3.3). The results indicate that the RMSEs are generally close to zero, supporting the validity of the proposed standard error estimators. The RMSEs for Kelley's true‐score estimators (ranging from .0001 to .0831) was generally larger than those for the SEM and the SEE (ranging from .0001 to .0079). For each of the estimators of interest, the Data S1 (Section 3.5) provide a scatterplot of the estimated and empirical standard errors. These plots also provide insight into the magnitude of the standard errors in the study.

**TABLE 3:** Summary statistics of the distribution of RMSE values of the proposed standard errors across the 96 study conditions.

Estimator	Equation	Min	Q1	Median	Mean	Q3	Max
$\hat{\tau }\left(\theta =-2\right)$	28	3	38	38	63	67	429
$\hat{\tau }\left(\theta =0\right)$	28	1	17	17	30	34	332
$\hat{\tau }\left(\theta =2\right)$	28	2	42	42	79	98	831
${\hat{\sigma }}_{E}$	29	1	2	5	6	10	17
${\hat{\sigma }}_{\epsilon }$	30	1	5	10	17	22	79

*Note*: All values were multiplied by 10,000 to improve readability.

Table 4 shows the distribution of the coverage rates of the Wald‐based 95% confidence intervals constructed using the standard error estimators across all 96 conditions (full tables are provided in the Data S1, Section 3.4). The results indicate that the coverage rates are generally close to 95%, supporting the validity of the proposed standard error estimators. For the smallest sample size (N=200) the average coverage rate was 94.0%, for (N=500) it was 94.5%, and for (N=2.000) it was 94.9%, indicating that for smaller samples, the standard errors are slightly liberal.

**TABLE 4:** Summary statistics of the coverage rate distribution of the proposed standard error estimators across the 96 study conditions.

Estimator	Equation	Min	Q1	Median	Mean	Q3	Max
$\hat{\tau }\left(\theta =-2\right)$	28	91.5	94.3	94.7	94.6	95.0	95.5
$\hat{\tau }\left(\theta =0\right)$	28	90.8	94.7	95.0	94.9	95.3	96.1
$\hat{\tau }\left(\theta =2\right)$	28	92.9	94.2	94.6	94.6	95.0	95.8
${\hat{\sigma }}_{E}$	29	93.6	94.7	94.9	94.9	95.1	95.6
${\hat{\sigma }}_{\epsilon }$	30	89.0	94.2	94.7	94.2	95.1	98.9

*Note*: Coverage rates are given in percentages. The estimated true scores were compared with $\alpha x+\left(1-\alpha \right)\mu_{X}$, the estimated SEM to $\sigma_{X}\sqrt{1-\alpha }$ and the estimated SEE to $\sigma_{X}\sqrt{\alpha \left(1-\alpha \right)}$.

#### 5.2.3 Conclusion

Based on these results, we found no evidence against the use of the proposed bias approximations and standard error estimators. In the absence of alternative bias approximations or standard error estimators for these parameters, we therefore recommend their use.

## 6 ACCURACY IN THE CLASSICAL METHOD AND KELLEY'S METHOD

Given the support for the bias approximations (Equations 20, 23 and 26) and the standard error estimators (Equations 27, 28 and 29), one may quantify the joint effect of bias and sampling variability on the accuracy of the parameter estimator $\hat{\lambda }$ using the RMSE; that is,

(31) $$\text{RMSE}=\sqrt{\tilde{\text{Bias}}{\left(\hat{\lambda }\right)}^{2}+\hat{\mathrm{SE}}{\left(\hat{\lambda }\right)}^{2}},$$

where $\tilde{\text{Bias}}{\left(\hat{\lambda }\right)}^{2}$ is the squared bias approximation, and $\hat{\mathrm{SE}}{\left(\hat{\lambda }\right)}^{2}=\hat{\mathrm{Var}}\left(\hat{\lambda }\right)$ is the squared estimated standard error.

For $\hat{\tau }\left(\theta =-2\right)$, $\hat{\tau }\left(\theta =0\right)$, $\hat{\tau }\left(\theta =2\right)$, ${\hat{\sigma }}_{E}$ and ${\hat{\sigma }}_{\epsilon }$, the RMSE was computed for all 96 conditions used in the simulation study. Table 5 shows the parameters and the RMSEs for four scenarios: the best‐case and worst‐case scenarios for N=200 and N=2000. The results for all 96 conditions are available in the Data S1 (Section 4). The difference in RMSE within the same scenario between N=200 and N=2000 reflects the effect of sampling variability, because the bias approximations are independent of sample size. The RMSE for Kelley's true‐score estimator can be large for extreme true scores. In addition, the RMSEs of the estimated SEM and SEE can be sizable.

**TABLE 5:** Accuracy of estimators for Kelley's true‐score, standard error of measurement (SEM) and standard error estimation (SEE): The best‐case and worst‐case scenario for small and large samples.

Estimator	Best‐case scenario			Worst‐case scenario		
	Parameter	RMSE		Parameter	RMSE	
		N=200	N=2000		N=200	N=2000
$\hat{\tau }\left(\theta =-2\right)$ ^a^	.0002	.1204	.1192	2.1978	1.5307	1.5215
$\hat{\tau }\left(\theta =0\right)$ ^b^	3.0000	.0303	.0096	5.3988	.2902	.1658
$\hat{\tau }\left(\theta =2\right)$ ^c^	5.9519	.2925	.2895	9.8022	1.5307	1.5215
${\hat{\sigma }}_{E}$ ^d^	1.6330	.0157	.0049	.6344	.3181	.3174
${\hat{\sigma }}_{\epsilon }$ ^e^	.8695	.0042	.0006	.6019	.2357	.2343

^a^ For $\hat{\tau }\left(\theta =-2\right)$, best‐case: J=6, $\rho_{X}=.9$. $\text{Bias}\;\left({\hat{\rho }}_{X}\right)=-.05$; worst‐case: J=12, $\rho_{X}=.6$. $\text{Bias}\;\left({\hat{\rho }}_{X}\right)=.00$.

^b^ For $\hat{\tau }\left(\theta =0\right)$, best case: J=6, $\rho_{X}=.9$. $\text{Bias}\;\left({\hat{\rho }}_{X}\right)=.00$; worst case: J=6, $\rho_{X}=.6$. $\text{Bias}\;\left({\hat{\rho }}_{X}\right)=-.05$.

^c^ For $\hat{\tau }\left(\theta =-2\right)$, best case: J=6, $\rho_{X}=.9$. $\text{Bias}\;\left({\hat{\rho }}_{X}\right)=-.02$; worst case: J=12, $\rho_{X}=.6$. $\text{Bias}\;\left({\hat{\rho }}_{X}\right)=.00$.

^d^ For ${\hat{\sigma }}_{E}$, best case: J=12, $\rho_{X}=.6$. $\text{Bias}\;\left({\hat{\rho }}_{X}\right)=.00$; worst case: J=12, $\rho_{X}=.9$. $\text{Bias}\;\left({\hat{\rho }}_{X}\right)=-.10$.

^e^ For ${\hat{\sigma }}_{\epsilon }$, best case: J=6, $\rho_{X}=.7$. $\text{Bias}\;\left({\hat{\rho }}_{X}\right)=.00$; worst case: J=12, $\rho_{X}=.9$. $\text{Bias}\;\left({\hat{\rho }}_{X}\right)=-.10$.

## 7 BIAS PROJECTION

The analytic bias approximations in Equations 20, 23 and 26 allow graphic display of the bias in the estimators for Kelley's true scores, the SEM and the SEE. Such graphs may help to assess the bias in samples. Figure 2 shows approximations (Equation 20) of the finite‐sample bias (right‐hand panel) and the total bias (i.e. finite‐sample bias and asymptotic bias; left‐hand panel) of Kelley's true‐score estimator. It follows from Equation 20 that unless $\tau_{v}^{*}=\tau_{v}$, which occurs when $\rho_{X}=1$ or $\rho_{X}=0$, the asymptotic bias will always be present and does not vanish as the sample size increases. The finite‐sample bias, $\text{Bias}\left({\hat{\rho }}_{X}\right)\left[\tau_{v}-\mu_{X}\right]$, depends on both the magnitude of the bias of the reliability estimator, and the discrepancy between a person's true score and the mean test score. In general, it is difficult to assess the bias of a reliability estimator without making assumptions about the data beyond CTT. For lower bounds to reliability, such as Cronbach's alpha, $\text{Bias}\left({\hat{\rho }}_{X}\right)<0$ (unless items or test parts would satisfy unrealistic conditions no data set would satisfy; Sijtsma & Pfadt, 2021), which makes $\text{Bias}\left({\hat{\rho }}_{X}\right)\left[\tau_{v}-\mu_{X}\right]$ positive for persons with true scores below average, and negative for persons with true scores above average.

**FIGURE 2:**
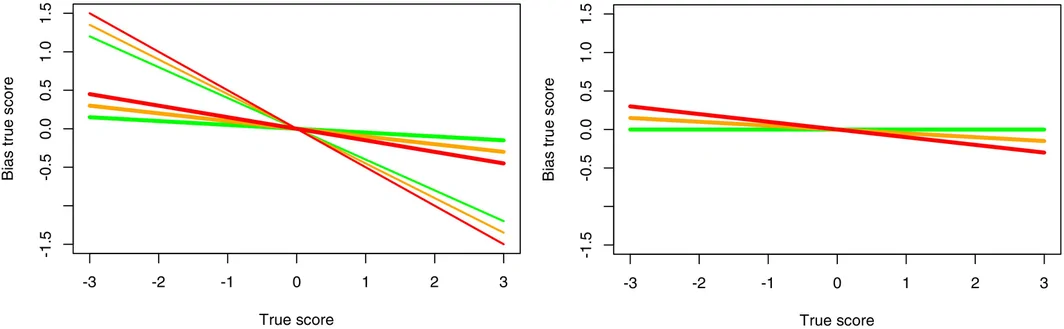
Approximate total bias (left) and approximate finite sample bias (right) of Kelley's true‐score estimates for standard normally distributed test scores, for two levels of reliability and three levels of reliability bias. Reliability levels are .9 (thick lines) and .6 (thin lines). Reliability‐bias levels are 0 (green), −.5 (orange) and − 10 (red). For the estimation bias (right), reliability has no effect, so the lines pertaining to the two reliability factors overlap.

For different levels of bias in the reliability estimator, Figure 3 shows approximations of the bias of the estimated SEM (left‐hand panel, Equation 23) and the estimated SEE (right‐hand panel, Equation 26). In practice, $\text{Bias}\left({\hat{\rho }}_{X}\right)$ is negative for lower bound estimators. As a result, $\text{Bias}\left({\hat{\sigma }}_{E}\right)$ will be positive, and the estimated SEM tends to overestimate the error component. Figure 3 (left‐hand panel) shows that $\text{Bias}\left({\hat{\sigma }}_{E}\right)$ increases when $\rho_{X}$ increases or the magnitude of $\text{Bias}\left({\hat{\rho }}_{X}\right)$ increases. Only if the reliability estimator is unbiased, the estimated SEM will be unbiased. Equation 26 shows that the bias approximation of the SEE estimator depends on the variance of the reliability estimator, the reliability itself and the bias of the reliability estimator. The reliability is present both in the numerator and denominator of Equation 26, and its complicated effect on effect $\text{Bias}\left({\hat{\sigma }}_{\epsilon }\right)$ is best displayed graphically (Figure 3, right‐hand panel). For lower bounds to the reliability with values that are deemed acceptable in psychological research (e.g. $\rho_{X}>.6$), the bias of the estimated SEE is typically positive, similar to the SEM. Thus, in practice, the SEE is a conservative estimator.

**FIGURE 3:**
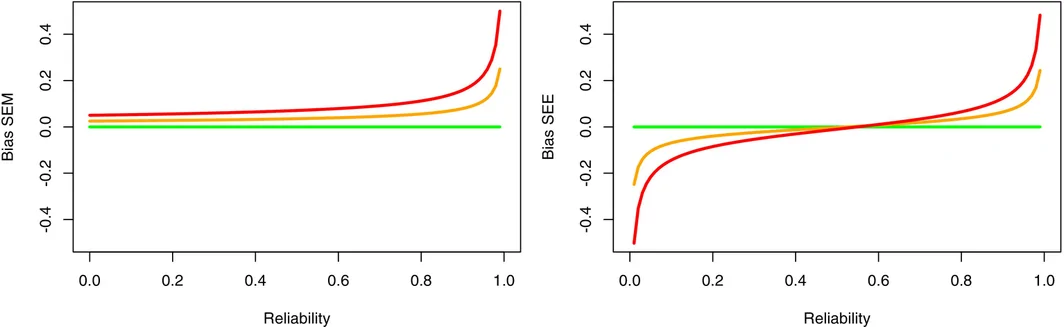
Approximate bias in the estimated standard error of measurement (SEM) (left) and estimated standard error estimation (SEE) (right), for standard normally distributed test scores, for different levels of the reliability (horizontal axis) and different levels of the bias in the reliability estimate. Reliability‐bias levels are: −.10 (red), −.05 (orange) and 0 (green).

## 8 DISCUSSION

CTT provides two approaches for estimating the true score, each accompanied by a measure of uncertainty. In the comparisons and discussions of these methods, the extent to which these methods are affected by estimation bias and sampling variability has been largely ignored. While the classical true‐score estimator is unbiased, we derived expressions for bias in Kelley's true‐score estimates and in the estimated SEM and SEE. By omitting covariances between parameter estimates and higher‐order variance terms, we derived bias approximations that allow researchers to examine the bias of the true score, SEM and SEE estimators across a range of plausible values for the reliability and its bias. Using simulations, we showed that these bias approximations can be used safely to examine bias prior to a study.

Two points should be noted regarding our definition of bias of Kelley's true‐score estimator. First, several authors (e.g. Holland & Hoskens, 2003) noted that Kelley's true‐score estimator is biased. However, these authors assumed known reliability values and therefore considered only the asymptotic bias, without taking into account the additional finite sample bias. Second, we defined its bias as $E\left({\hat{\tau }}_{v}^{K}\right)-\tau_{v}$, (Equation 19), whereas one could argue that it could also be defined as $E\left({\hat{\tau }}_{v}^{K}\right)-\tau_{v}^{*}$, which eliminates the asymptotic bias. However, using this alternative definition of bias has two disadvantages. First, the two methods would no longer estimate the same parameter, and more importantly, Kelley's true‐score estimator would lose its property as BLP.

To investigate sampling variability, we provided estimators for the standard errors of Kelley's true‐score estimate, the SEM estimate and the SEE estimate under a multinomial sampling scheme. As a multinomial sample scheme is very flexible, these estimators can be applied irrespective of the distribution of the test data. These estimators treat each unique item‐score pattern observed in the data as a separate category in a multinomial distribution. On regular laptops, memory problems may occur if the number of categories exceeds 1,000,000. Because unobserved item‐score patterns do not contribute to the standard errors and can therefore be ignored (e.g. Kuijpers et al., 2013), the number of categories cannot exceed N. Consequently, estimation remains feasible even for a large number of items. However, if the sample size is too small to accurately estimate the variances and covariances of the sample covariance matrix, which are required to estimate the standard errors of the lower bound reliability estimates, the resulting standard errors may be inaccurate. Also, if the number of response categories becomes large (e.g. semi‐continuous responses), the procedure becomes slow (e.g. see Van der Ark, 2025).

Simulations showed that the estimated standard errors were accurate under wide variety of conditions and even for reasonably small samples (N=200). Simulations further indicated that the effect of sampling variability is usually small. It should be noted that in our simulation study, we used item parameters of the two‐parameter logistic model that yielded specific values of both $\rho_{X}$ and Bias(${\hat{\rho }}_{X}$). However, there may be other sets of item parameters that yield the same values of $\rho_{X}$ and Bias(${\hat{\rho }}_{X}$), but different values for $\mu_{X}$ and $\sigma_{X}^{2}$, and subsequently different values for $\sigma_{Τ}^{2}$, ${\tilde{\tau }}_{v}$, $\sigma_{E}^{2}$ and $\sigma_{\epsilon }^{2}$ and their estimates. Hence, the values the true scores, SEM, SEE are test specific. The results of our simulations should therefore primarily be regarded as a validation of the analytical results, and as providing insight into general trends in bias and standard errors one may expect.

## DISCLOSURE OF ARTIFICIAL INTELLIGENCE–GENERATED CONTENT (AIGC) TOOLS

The authors used ChatGPT (OpenAI, 2025) to improve spelling, grammar and general editing. The authors reviewed the content generated and take full responsibility for the content of the article.

## AUTHOR CONTRIBUTIONS

**L. Andries van der Ark:** Conceptualization; investigation; writing – original draft; methodology; writing – review and editing; software; formal analysis; project administration; visualization; validation. **Wilco H. M. Emons:** Conceptualization; investigation; writing – original draft; methodology; writing – review and editing; software; formal analysis; validation. **Jules L. Ellis:** Investigation; formal analysis; writing – review and editing; methodology. **Klaas Sijtsma:** Conceptualization; investigation; writing – original draft; formal analysis; writing – review and editing; methodology.

## CONFLICT OF INTEREST STATEMENT

We have no known conflict of interest to disclose.

## Supporting information


Data S1.

## Data Availability

All code and data used in this paper are available from OSF and accessible to the editor and the reviewers via https://osf.io/v2psk/.

## APPENDIX A BIAS OF THE TRUE‐SCORE ESTIMATOR IN THE CLASSICAL METHOD

For ${\hat{\tau }}_{v}^{C}=X_{v}$ (Equation 5), bias is defined as $\text{Bias}\left({\hat{\tau }}_{v}^{C}\right)=E\left({\hat{\tau }}_{v}^{C}\right)-\tau_{v}.$ Under the CTT, where $E\left(X_{v}\right)=\tau_{v}$ (Equation 1), the bias equals

(A1)
$$\text{Bias}\left({\hat{\tau }}_{v}^{C}\right)=E\left({\hat{\tau }}_{v}^{C}\right)-\tau_{v}=E\left(X_{v}\right)-\tau_{v}=\tau_{v}-\tau_{v}=0.$$

## APPENDIX B BIAS OF THE TRUE‐SCORE ESTIMATOR IN KELLEY'S METHOD

The finite‐sample bias in Equation (16) equals

(B1)
$$\begin{array}{cc} E\left({\hat{\tau }}_{v}^{K}\right)-\tau_{v}^{*} & =E\left[{\hat{\rho }}_{X}X_{v}+\left(1-{\hat{\rho }}_{X}\right){\hat{\mu }}_{X}\right]-\left[\rho_{X}\tau_{v}+\left(1-\rho_{X}\right)\mu_{X}\right]\\ & =E\left({\hat{\rho }}_{X}X_{v}\right)+E\left({\hat{\mu }}_{X}\right)-E\left({\hat{\rho }}_{X}{\hat{\mu }}_{X}\right)-\left[\rho_{X}\tau_{v}+\mu_{X}-\rho_{X}\mu_{X}\right]\\ & =E\left({\hat{\rho }}_{X}\right)E\left(X_{v}\right)+\mathrm{Cov}\left({\hat{\rho }}_{X},X_{v}\right)+E\left({\hat{\mu }}_{X}\right)-E\left({\hat{\rho }}_{X}\right)E\left({\hat{\mu }}_{X}\right)+\mathrm{Cov}\left({\hat{\rho }}_{X},{\hat{\mu }}_{X}\right)-\rho_{X}\tau_{v}-\mu_{X}+\rho_{X}\mu_{X}. \end{array}$$

As the sample mean is an unbiased estimator of $\mu_{X}$ and $E\left(X_{v}\right)=\tau_{v}$, Equation B1 reduces to

(B2)
$$\begin{array}{cc} E\left({\hat{\tau }}_{v}^{K}\right)-\tau_{v}^{*} & =E\left({\hat{\rho }}_{X}\right)\tau_{v}+\mathrm{Cov}\left({\hat{\rho }}_{X},X_{v}\right)-E\left({\hat{\rho }}_{X}\right)\mu_{X}+\mathrm{Cov}\left({\hat{\rho }}_{X},{\hat{\mu }}_{X}\right)-\rho_{X}\tau_{v}+\rho_{X}\mu_{X}\\ & =\left[E\left({\hat{\rho }}_{X}\right)-\rho_{X}\right]\tau_{v}-\left[E\left({\hat{\rho }}_{X}\right)-\rho_{X}\right]\mu_{X}+\mathrm{Cov}\left({\hat{\rho }}_{X},X_{v}\right)+\mathrm{Cov}\left({\hat{\rho }}_{X},{\hat{\mu }}_{X}\right)\\ & =\text{Bias}\left({\hat{\rho }}_{X}\right)\tau_{v}-\text{Bias}\left({\hat{\rho }}_{X}\right)\mu_{X}+\mathrm{Cov}\left({\hat{\rho }}_{X},X_{v}\right)+\mathrm{Cov}\left({\hat{\rho }}_{X},{\hat{\mu }}_{X}\right)\\ & =\text{Bias}\left({\hat{\rho }}_{X}\right)\left(\tau_{v}-\mu_{X}\right)+\mathrm{Cov}\left({\hat{\rho }}_{X},X_{v}\right)+\mathrm{Cov}\left({\hat{\rho }}_{X},{\hat{\mu }}_{X}\right), \end{array}$$

where the last term equals the right‐hand side of Equation 18.

Under some conditions, the covariance terms in Equation B2 may equal 0. If person v's test score was not involved in the estimation of the reliability, then $\mathrm{Cov}\left({\hat{\rho }}_{X},X_{v}\right)=0$. This happens often in practice, when psychological researchers use a validated test for their research, and the reliability was estimated in a different norm sample. If person v's test was involved in the estimation of the reliability, then $\mathrm{Cov}\left({\hat{\rho }}_{X},X_{v}\right)\to 0$ if N→∞. Hence, for large samples, the effect of $\mathrm{Cov}\left({\hat{\rho }}_{X},X_{v}\right)$ on $E\left({\hat{\tau }}_{v}^{K}\right)$ is small. Otherwise, $\mathrm{Cov}\left({\hat{\rho }}_{X},X_{v}\right)$ is generally not equal to zero.

If person the mean and the reliability were estimated in different samples, then $\mathrm{Cov}\left({\hat{\rho }}_{X},{\hat{\mu }}_{X}\right)=0.$ Also, if the item scores follow a multivariate normal distribution, then mean vector $\hat{\mu }$ and covariance matrix $\hat{\Sigma }$ are independent. Now, if the mean test score is a function of $\hat{\mu }$ [i.e. ${\hat{\mu }}_{X}=f\left(\hat{\mu }\right)$] and if the reliability estimate is a function of $\hat{\Sigma }$ [i.e. ${\hat{\rho }}_{X}=f\left(\hat{\Sigma }\right)$], then $\mathrm{Cov}\left({\hat{\rho }}_{X},{\hat{\mu }}_{X}\right)=0$. If the test score is the weighted or unweighted sum or mean of the item scores, with weights independent of the covariances—which is typically the case in when test constructors apply CTT—then ${\hat{\mu }}_{X}=f\left(\hat{\mu }\right)$. ${\hat{\rho }}_{X}=f\left(\hat{\Sigma }\right)$ is true for all lower bound estimates of the reliability, such as Cronbach's alpha and Guttman's lambda series.

## APPENDIX C BIAS OF THE ESTIMATED SEM

First, consider the bias of the squared SEM. For ${\hat{\sigma }}_{E}^{2}={\hat{\sigma }}_{X}^{2}\;\left(1-{\hat{\rho }}_{X}\right)$(cf. Equation 11),

(C1)
$$\begin{array}{cc} \text{Bias}\left({\hat{\sigma }}_{E}^{2}\right) & =E\left[{\hat{\sigma }}_{X}^{2}\;\left(1-{\hat{\rho }}_{X}\right)\right]-\sigma_{X}^{2}\left(1-\rho_{X}\right)\\ & =E\left({\hat{\sigma }}_{X}^{2}\right)-E\left({\hat{\sigma }}_{X}^{2}{\hat{\rho }}_{X}\right)-\sigma_{X}^{2}+\sigma_{X}^{2}\rho_{X}\\ & =E\left({\hat{\sigma }}_{X}^{2}\right)-E\left({\hat{\sigma }}_{X}^{2}\right)E\left({\hat{\rho }}_{X}\right)+\mathrm{Cov}\left({\hat{\sigma }}_{X}^{2},{\hat{\rho }}_{X}\right)-\sigma_{X}^{2}+\sigma_{X}^{2}\rho_{X}. \end{array}$$

Assuming that ${\hat{\sigma }}_{X}^{2}$ is the unbiased variance estimator, so $E\left({\hat{\sigma }}_{X}^{2}\right)=\sigma_{X}^{2}$, Equation C1 reduces to

(C2)
$$\begin{array}{cc} \text{Bias}\left({\hat{\sigma }}_{E}^{2}\right) & =\sigma_{X}^{2}-\sigma_{X}^{2}E\left({\hat{\rho }}_{X}\right)-\sigma_{X}^{2}+\sigma_{X}^{2}\rho_{X}+\mathrm{Cov}\left({\hat{\sigma }}_{X}^{2},{\hat{\rho }}_{X}\right)\\ & =-\sigma_{X}^{2}E\left({\hat{\rho }}_{X}\right)+\sigma_{X}^{2}\rho_{X}+\mathrm{Cov}\left({\hat{\sigma }}_{X}^{2},{\hat{\rho }}_{X}\right)\\ & =-\sigma_{X}^{2}\left[\text{Bias}\left({\hat{\rho }}_{X}\right)\right]+\mathrm{Cov}\left({\hat{\sigma }}_{X}^{2},{\hat{\rho }}_{X}\right). \end{array}$$

The last term of Equation C2 equals Equation 21 in the main text.

In general, the covariance term in Equation C2, $\mathrm{Cov}\left({\hat{\sigma }}_{X}^{2},{\hat{\rho }}_{X}\right)$, does not equal zero. If ${\hat{\sigma }}_{X}^{2}$ and ${\hat{\rho }}_{X}$ are estimated from different samples, which occurs, for example, if researchers adopt the reliability estimate from the test manual, and compute the test‐score variance from their own data, $\mathrm{Cov}\left({\hat{\sigma }}_{X}^{2},{\hat{\rho }}_{X}\right)=0$.

To find the bias of the estimated SEM, we used a second‐order Taylor expansion around $\sigma_{E}^{2}$. Let $g\left(x\right)=\sqrt{x}$, with $g^{\prime }\left(x\right)=\frac{1}{2\sqrt{x}}$, and $g^{\prime \prime }\left(x\right)=-\frac{1}{4x^{3/2}}$. Then,

(C3)
$$\begin{array}{cc} E\left[{\hat{\sigma }}_{E}\right] & =E\left[g\left({\hat{\sigma }}_{E}^{2}\right)\right]\approx g\left(\sigma_{E}^{2}\right)+g^{\prime }\left(\sigma_{E}^{2}\right)\text{Bias}\left({\hat{\sigma }}_{E}^{2}\right)+\frac{1}{2}g^{\prime \prime }\left(\sigma_{E}^{2}\right)\mathrm{Var}\left({\hat{\sigma }}_{E}^{2}\right)\\ & =\sigma_{E}+\frac{1}{2\sigma_{E}}\text{Bias}\left({\hat{\sigma }}_{E}^{2}\right)-\frac{1}{8\sigma_{E}^{3}}\mathrm{Var}\left({\hat{\sigma }}_{E}^{2}\right). \end{array}$$

Using Equation C3, it then follows that:

(C4)
$$\begin{array}{cc} \text{Bias}\left({\hat{\sigma }}_{E}\right) & =E\left({\hat{\sigma }}_{E}\right)-\sigma_{E}\approx \hat{\text{Bias}}\left({\hat{\sigma }}_{E}\right)=\sigma_{E}+\frac{1}{2\sigma_{E}}\text{Bias}\left({\hat{\sigma }}_{E}^{2}\right)-\frac{1}{8\sigma_{E}^{3}}\mathrm{Var}\left({\hat{\sigma }}_{E}^{2}\right)-\sigma_{E}\\ & =\frac{1}{2\sigma_{E}}\left\{-\sigma_{X}^{2}\left[\text{Bias}\left({\hat{\rho }}_{X}\right)\right]+\mathrm{Cov}\left({\hat{\sigma }}_{X}^{2},{\hat{\rho }}_{X}\right)\right\}-\frac{1}{8\sigma_{E}^{3}}\mathrm{Var}\left({\hat{\sigma }}_{E}^{2}\right). \end{array}$$

Replacing $\sigma_{E}$ by $\sigma_{X}\sqrt{1-\rho_{X}}$ yields

(C5)
$$\hat{\text{Bias}}\left({\hat{\sigma }}_{E}\right)=\frac{1}{2\sigma_{X}\sqrt{1-\rho_{X}}}\left\{-\sigma_{X}^{2}\left[\text{Bias}\left({\hat{\rho }}_{X}\right)\right]+\mathrm{Cov}\left({\hat{\sigma }}_{X}^{2},{\hat{\rho }}_{X}\right)\right\}-\frac{1}{8\sigma_{E}^{3}}\mathrm{Var}\left({\hat{\sigma }}_{E}^{2}\right).$$

Note that Equation C5 equals Equation 22 in the main text.

$\mathrm{Var}\left({\hat{\sigma }}_{E}^{2}\right)$ in the last term of Equation C5 can be approximated: $\mathrm{Var}\left({\hat{\sigma }}_{E}^{2}\right)\approx 4\sigma_{E}\mathrm{Var}\left({\hat{\sigma }}_{E}\right)+2\mathrm{Var}{\left({\hat{\sigma }}_{E}\right)}^{2}$. Equation 29 provides an estimator for $\mathrm{Var}\left({\hat{\sigma }}_{E}\right)$, and $\sigma_{E}$ may be estimated using Equation 10. For large samples, the term $-\frac{1}{8\sigma_{E}^{3}}\mathrm{Var}\left({\hat{\sigma }}_{E}^{2}\right)$ vanishes.

As an aside, $\text{Bias}\left({\hat{\sigma }}_{E}^{2}\right)$ can also be written in terms of $\text{Bias}\left({\hat{\sigma }}_{T}^{2}\right)$. First, all reliability estimators can be written in the form

(C6)
$${\hat{\rho }}_{X}=\frac{{\hat{\sigma }}_{T}^{2}}{{\hat{\sigma }}_{X}^{2}}.$$

Because both ${\hat{\rho }}_{X}$ and ${\hat{\sigma }}_{X}^{2}$ can be computed from the test data, ${\hat{\sigma }}_{T}^{2}$ may be defined as ${\hat{\sigma }}_{T}^{2}={\hat{\rho }}_{X}{\hat{\sigma }}_{X}^{2}$. It follows from Equation 5 that ${\hat{\sigma }}_{E}^{2}={\hat{\sigma }}_{X}^{2}-{\hat{\sigma }}_{Τ}^{2}$. As a result, $\text{Bias}\left({\hat{\sigma }}_{E}^{2}\right)$ can be written as

(C7)
$$\begin{array}{cc} \text{Bias}\left({\hat{\sigma }}_{E}^{2}\right) & =E\left({\hat{\sigma }}_{E}^{2}\right)-\sigma_{E}^{2}=E\left({\hat{\sigma }}_{X}^{2}-{\hat{\sigma }}_{Τ}^{2}\right)-\left(\sigma_{X}^{2}-\sigma_{Τ}^{2}\right)\\ & =E\left({\hat{\sigma }}_{X}^{2}\right)-E\left({\hat{\sigma }}_{Τ}^{2}\right)-\sigma_{X}^{2}+\sigma_{Τ}^{2}=E\left({\hat{\sigma }}_{Τ}^{2}\right)-\sigma_{Τ}^{2}=-\text{Bias}\left({\hat{\sigma }}_{Τ}^{2}\right). \end{array}$$

## APPENDIX D BIAS OF THE ESTIMATED SEE

As in Appendix C, we first consider the bias of the squared SEE. For ${\hat{\sigma }}_{\epsilon }^{2}={\hat{\sigma }}_{X}^{2}\;{\hat{\rho }}_{X}\left(1-{\hat{\rho }}_{X}\right)$ (cf. Equation 16),

(D1)
$$\begin{array}{cc} \text{Bias}\left({\hat{\sigma }}_{\epsilon }^{2}\right) & =E\left[{\hat{\sigma }}_{X}^{2}\;{\hat{\rho }}_{X}\left(1-{\hat{\rho }}_{X}\right)\right]-\sigma_{X}^{2}\rho_{X}\left(1-\rho_{X}\right)\\ & =E\left[{\hat{\sigma }}_{X}^{2}\;{\hat{\rho }}_{X}\right]-E\left[{\hat{\sigma }}_{X}^{2}\;{\hat{\rho }}_{X}^{2}\right]-\sigma_{X}^{2}\rho_{X}+\sigma_{X}^{2}\rho_{X}^{2}\\ & =E\left[{\hat{\sigma }}_{X}^{2}\;\right]E\left[{\hat{\rho }}_{X}\right]+\mathrm{Cov}\left({\hat{\sigma }}_{X}^{2},{\hat{\rho }}_{X}\right)-E\left[{\hat{\sigma }}_{X}^{2}\;\right]E\left[{\hat{\rho }}_{X}^{2}\right]-\mathrm{Cov}\left({\hat{\sigma }}_{X}^{2},{\hat{\rho }}_{X}^{2}\right)-\sigma_{X}^{2}\rho_{X}+\sigma_{X}^{2}\rho_{X}^{2}. \end{array}$$

Assuming that ${\hat{\sigma }}_{X}^{2}$ is the unbiased variance estimator, so $E\left({\hat{\sigma }}_{X}^{2}\right)=\sigma_{X}^{2}$, and combining the two covariance terms, Equation D1 reduces to

(D2)
$$\begin{array}{c} =\sigma_{X}^{2}E\left[{\hat{\rho }}_{X}\right]+\mathrm{Cov}\left({\hat{\sigma }}_{X}^{2},{\hat{\rho }}_{X}-{\hat{\rho }}_{X}^{2}\right)-\sigma_{X}^{2}E\left[{\hat{\rho }}_{X}^{2}\right]-\mathrm{Cov}\left({\hat{\sigma }}_{X}^{2}\right)-\sigma_{X}^{2}\rho_{X}+\sigma_{X}^{2}\rho_{X}^{2}\\ =\sigma_{X}^{2}\left[\text{Bias}\left({\hat{\rho }}_{X}\right)-\text{Bias}\left({\hat{\rho }}_{X}^{2}\right)\right]+\mathrm{Cov}\left({\hat{\sigma }}_{X}^{2},{\hat{\rho }}_{X}-{\hat{\rho }}_{X}^{2}\right). \end{array}$$

The term Bias $\left({\hat{\rho }}_{X}^{2}\right)$ in Equation D2 can be further elaborated:

(D3)
$$\begin{array}{cc} \text{Bias}\;\left({\hat{\rho }}_{X}^{2}\right) & =E\left({\hat{\rho }}_{X}^{2}\right)-\rho_{X}^{2}\\ & =\mathrm{Var}\left({\hat{\rho }}_{X}\right)+{\left[E\left({\hat{\rho }}_{X}\right)\right]}^{2}-\rho_{X}^{2}\\ & =\mathrm{Var}\left({\hat{\rho }}_{X}\right)+{\left[\rho_{X}+\text{Bias}\left({\hat{\rho }}_{X}\right)\right]}^{2}-\rho_{X}^{2}\\ & =\mathrm{Var}\left({\hat{\rho }}_{X}\right)+2\rho_{X}\text{Bias}\left({\hat{\rho }}_{X}\right)+{\left[\text{Bias}\left({\hat{\rho }}_{X}\right)\right]}^{2}. \end{array}$$

Then, substituting Equation D3 into Equation D2 yields.

(D4)
$$\begin{array}{cc} \text{Bias}\left({\hat{\sigma }}_{\epsilon }^{2}\right) & =\sigma_{X}^{2}\left[\text{Bias}\left({\hat{\rho }}_{X}\right)-\mathrm{Var}\left({\hat{\rho }}_{X}\right)-2\rho_{X}\text{Bias}\left({\hat{\rho }}_{X}\right)-\text{Bias}{\left({\hat{\rho }}_{X}\right)}^{2}\right]+\mathrm{Cov}\left({\hat{\sigma }}_{X}^{2},{\hat{\rho }}_{X}-{\hat{\rho }}_{X}^{2}\right)\\ & =\sigma_{X}^{2}\left[-\text{Bias}{\left({\hat{\rho }}_{X}\right)}^{2}+\left(1-2\rho_{X}\right)\text{Bias}\left({\hat{\rho }}_{X}\right)-\mathrm{Var}\left({\hat{\rho }}_{X}\right)\right]. \end{array}$$

Equation D4 equals Equation 24 in the main text. Note that $\mathrm{Cov}\left({\hat{\sigma }}_{X}^{2},{\hat{\rho }}_{X}-{\hat{\rho }}_{X}^{2}\right)=0$ if ${\hat{\sigma }}_{X}^{2}$ and ${\hat{\rho }}_{X}$ are estimated from different samples, which occurs, for example, if researchers adopt the reliability estimate from the test manual, and compute the test‐score variance from their own data. But in general, $\mathrm{Cov}\left({\hat{\sigma }}_{X}^{2},{\hat{\rho }}_{X}-{\hat{\rho }}_{X}^{2}\right)\neq 0$.

To find the bias of the estimated SEE, we used a second‐order Taylor expansion around $\sigma_{\epsilon }^{2}$. Let $g\left(x\right)=\sqrt{x}$. With $g^{\prime }\left(x\right)=\frac{1}{2\sqrt{x}}$, and $g^{\prime \prime }\left(x\right)=-\frac{1}{4x^{3/2}}$. Then,

(D5)
$$\begin{array}{c} E\left[g\left({\hat{\sigma }}_{\epsilon }^{2}\right)\right]\approx g\left(\sigma_{\epsilon }^{2}\right)+g^{\prime }\left(\sigma_{\epsilon }^{2}\right)\text{Bias}\left({\hat{\sigma }}_{\epsilon }^{2}\right)+\frac{1}{2}g^{\prime \prime }\left(\sigma_{\epsilon }^{2}\right)\mathrm{Var}\left({\hat{\sigma }}_{\epsilon }^{2}\right)\\ =\sigma_{\epsilon }+\frac{1}{2\sigma_{\epsilon }}\text{Bias}\left({\hat{\sigma }}_{\epsilon }^{2}\right)-\frac{1}{8\sigma_{\epsilon }^{3}}\mathrm{Var}\left({\hat{\sigma }}_{\epsilon }^{2}\right). \end{array}$$

It then follows:

(D6)
$$\begin{array}{cc} \text{Bias}\left({\hat{\sigma }}_{\epsilon }\right) & =E\left({\hat{\sigma }}_{\epsilon }\right)-\sigma_{\epsilon }\\ \approx \hat{\text{Bias}}\left({\hat{\sigma }}_{\epsilon }\right) & =\sigma_{\epsilon }+\frac{1}{2\sigma_{\epsilon }}\text{Bias}\left({\hat{\sigma }}_{\epsilon }^{2}\right)-\frac{1}{8\sigma_{\epsilon }^{3}}\mathrm{Var}\left({\hat{\sigma }}_{\epsilon }^{2}\right)-\sigma_{\epsilon }\\ & =\frac{\sigma_{X}^{2}\left[-\text{Bias}{\left({\hat{\rho }}_{X}\right)}^{2}+\left(1-2\rho_{X}\right)\text{Bias}\left({\hat{\rho }}_{X}\right)-\mathrm{Var}\left({\hat{\rho }}_{X}\right)\right]+\mathrm{Cov}\left({\hat{\sigma }}_{X}^{2},{\hat{\rho }}_{X}-{\hat{\rho }}_{X}^{2}\right)}{2\sigma_{\epsilon }}-\frac{1}{8\sigma_{\epsilon }^{3}}\mathrm{Var}\left({\hat{\sigma }}_{\epsilon }^{2}\right)\\ & =\frac{\sigma_{X}\left[-\text{Bias}{\left({\hat{\rho }}_{X}\right)}^{2}+\left(1-2\rho_{X}\right)\text{Bias}\left({\hat{\rho }}_{X}\right)-\mathrm{Var}\left({\hat{\rho }}_{X}\right)\right]+\mathrm{Cov}\left({\hat{\sigma }}_{X}^{2},{\hat{\rho }}_{X}-{\hat{\rho }}_{X}^{2}\right)}{2\sqrt{\rho_{X}\left(1-\rho_{X}\right)}}-\frac{1}{8\sigma_{\epsilon }^{3}}\mathrm{Var}\left({\hat{\sigma }}_{\epsilon }^{2}\right). \end{array}$$

## APPENDIX E DESIGN MATRICES AND FUNCTIONS FOR THE RECURSIVE COMPUTATION OF COEFFICIENTS ${\hat{\tau }}^{K}$, ${\hat{\sigma }}_{E}$ AND ${\hat{\sigma }}_{\epsilon }$ AND THEIR JACOBIANS

**TABLE E1:** Design matrices used for the recursive computation of coefficients ${\hat{\tau }}^{K}$, ${\hat{\sigma }}_{E}$ and ${\hat{\sigma }}_{\epsilon }$ and their Jacobians (see Table E2).

Coefficient	Design matrices		
All	$A_{1}=\left(\begin{array}{l} R\\ s\\ R^{\left(2\right)}\\ s^{\left(2\right)}\\ {u_{J}}^{\prime }\\ {u_{J}}^{\prime } \end{array}\right)$	$A_{2}=\left(\begin{array}{rrrrrr} 2I_{J} & o_{J} & O_{J} & o_{J} & o_{J} & o_{J}\\ {o_{J}}^{\prime } & 2 & {o_{J}}^{\prime } & 0 & 0 & 0\\ O_{J} & o_{J} & I_{J} & o_{J} & u_{J} & o_{J}\\ {o_{J}}^{\prime } & 0 & {o_{J}}^{\prime } & 1 & 1 & 0\\ {o_{J}}^{\prime } & 1 & {o_{J}}^{\prime } & 0 & -1 & 0\\ {o_{J}}^{\prime } & 0 & {o_{J}}^{\prime } & 0 & 1 & 0\\ {o_{J}}^{\prime } & 0 & {o_{J}}^{\prime } & 0 & 1 & -1 \end{array}\right)$	
	$A_{3}=\left(\begin{array}{rrrrrrr} -{u_{J}}^{\prime } & 0 & {u_{J}}^{\prime } & 0 & 0 & 0 & 0\\ {o_{J}}^{\prime } & -1 & {o_{J}}^{\prime } & 1 & 0 & 0 & 0\\ {o_{J}}^{\prime } & 0 & {o_{J}}^{\prime } & 0 & 1 & 0 & 0\\ {o_{J}}^{\prime } & 0 & {o_{J}}^{\prime } & 0 & 0 & 1 & 0\\ {o_{J}}^{\prime } & 0 & {o_{J}}^{\prime } & 0 & 0 & 1 & -1\\ {o_{J}}^{\prime } & 0 & {o_{J}}^{\prime } & 0 & 0 & 0 & 1 \end{array}\right)$	$A_{4}=\left(\begin{array}{rrrrrr} 1 & -1 & 0 & 0 & 0 & 0\\ 0 & 1 & 0 & -1 & -1 & 0\\ 0 & 0 & 1 & 0 & 0 & 0\\ 0 & 0 & 0 & 0 & 0 & 1 \end{array}\right)$	
${\hat{T}}^{K}$	$A_{5}=\left(\begin{array}{rrrr} -\frac{J}{J-1} & 0 & 0 & \frac{J}{J-1}\\ 0 & 0 & 1 & 0\\ o_{K} & o_{K} & o_{K} & x_{K} \end{array}\right).\left(\begin{array}{rrrr} 1 & 0 & 0 & {0_{K}}^{\prime }\\ 0 & 1 & 0 & {0_{K}}^{\prime }\\ -1 & 0 & 1 & {0_{K}}^{\prime }\\ o_{K} & o_{K} & o_{K} & I_{K} \end{array}\right)$		
	$A_{6}=\left(\begin{array}{rrrr} u_{K} & o_{K} & o_{K} & I_{K}\\ 0 & 1 & 1 & {o_{K}}^{\prime } \end{array}\right)$	$A_{7}=\left(I_{K}\;u_{K}\right)$	$A_{8}=I_{K}$
${\hat{\sigma }}_{E}$	$A_{5}^{*}=\left(\begin{array}{rrrr} -\frac{J}{J-1} & 0 & 0 & \frac{J}{J-1}\\ 0 & 1 & 0 & 0\\ 0 & 0 & 0 & 1 \end{array}\right).\left(\begin{array}{rrr} -1 & 0 & 1\\ 0 & 1 & 0 \end{array}\right)$	$A_{6}^{*}=\left(\frac{1}{2}\;\frac{1}{2}\right)$	
	$A_{5}^{†}=\left(\begin{array}{rrrr} -\frac{J}{J-1} & 0 & 0 & \frac{J}{J-1}\\ 0 & 1 & 0 & 0\\ 0 & 0 & 0 & 1 \end{array}\right).\left(\begin{array}{rrr} 1 & 0 & 0\\ -1 & 0 & 1\\ 0 & 1 & 0 \end{array}\right)$	$A_{6}^{†}=\left(\frac{1}{2}\;\frac{1}{2}\;\frac{1}{2}\right)$	
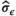

*Note*: $I_{H}$, Identity matrix of size H; J, Number of items; K, number of sum scores; L, Number of observed response patterns; $o_{H}$, vector of zeroes of length H; R, J×L matrix of observed response patterns; $R^{\left(2\right)}$, J×L matrix containing the squared elements of R; $s=u_{J}$ **R**, 1×L row vector containing the sum scores for each response pattern; $s^{\left(2\right)}$ 1×L row vector containing the squared elements of s; $u_{H}$, vector of ones of length H. $X^{\prime }$ denotes the transpose of X. A dot denotes the matrix product.

**TABLE E2:** Functions for the recursive computation of ${\hat{\tau }}^{K}$,${\hat{\sigma }}_{E}$ and ${\hat{\sigma }}_{\epsilon }$ (second column), and functions for the recurve computation of the Jacobian of ${\hat{\tau }}^{K}$,${\hat{\sigma }}_{E}$ and ${\hat{\sigma }}_{\epsilon }$ (third column).

Coefficient	Functions for computing coefficient	Functions for computing Jacobian
All	$g_{0}=n$	$G_{0}=I$
	$g_{1}=\log\left[A_{1}g_{0}\right]$	$G_{1}=D{\left[A_{1}g_{0}\right]}^{-1}A_{1}G_{0}$
	$g_{2}=\exp\;\left[A_{2}g_{1}\right]$	$G_{2}=D\left[\exp\;\left[A_{2}g_{1}\right]\right]A_{2}G_{1}$
	$g_{3}=\log\left[A_{3}g_{2}\right]$	$G_{3}=D{\left[A_{3}g_{2}\right]}^{-1}A_{3}G_{3}$
	$g_{4}=\exp\;\left[A_{4}g_{3}\right]$	$G_{4}=D\left[\exp\;\left[A_{4}g_{3}\right]\right]A_{4}G_{3}$
${\hat{\tau }}^{K}$	$g_{5}=\log\left[A_{5}g_{4}\right]$	$G_{5}=D{\left[A_{5}g_{4}\right]}^{-1}A_{5}G_{4}$
	$g_{6}=\exp\;\left[A_{6}g_{5}\right]$	$G_{6}=D\left[\exp\;\left[A_{6}g_{5}\right]\right]A_{6}G_{5}$
	$g_{7}=\log\left[A_{7}g_{6}\right]$	$G_{7}=D{\left[A_{7}g_{6}\right]}^{-1}A_{7}G_{6}$
	$g_{{\hat{\tau }}^{K}}=g_{8}=\exp\;\left[A_{8}g_{7}\right]$	$G_{{\hat{\tau }}^{K}}=G_{8}=D\left[\exp\;\left[A_{8}g_{7}\right]\right]A_{8}G_{7}$
${\hat{\sigma }}_{E}$	$g_{5}^{*}=\log\left[A_{5}^{*}g_{4}\right]$	$G_{5}^{*}=D{\left[A_{5}^{*}g_{4}\right]}^{-1}A_{5}^{*}G_{4}$
	$g_{{\hat{\sigma }}_{E}}=g_{6}^{*}=\exp\;\left[A_{6}^{*}g_{5}^{*}\right]$	$G_{{\hat{\sigma }}_{E}}=G_{6}^{*}=D\left[\exp\;\left[A_{6}^{*}g_{5}^{*}\right]\right]A_{6}^{*}G_{5}^{*}$
${\hat{\sigma }}_{\epsilon }$	$g_{5}^{†}=\log\left[A_{5}^{†}g_{4}\right]$	$G_{5}^{†}=D{\left[A_{5}^{†}g_{4}\right]}^{-1}A_{5}^{†}G_{4}$
	$g_{{\hat{\sigma }}_{\epsilon }}=g_{6}^{†}=\exp\;\left[A_{6}^{†}g_{5}^{†}\right]$	$G_{{\hat{\sigma }}_{\epsilon }}=G_{6}^{†}=D\left[\exp\;\left[A_{6}^{†}g_{5}^{†}\right]\right]A_{6}^{†}G_{5}^{†}$

*Note*: n is the vector of frequencies of the observed response patterns. For the values of design matrices $A_{1},\ldots$, $A_{8}$, $A_{5}^{*}$, $A_{6}^{*}$, $A_{5}^{†},A_{6}^{†}$, see Table E1. $D\left(x\right)$ is a diagonal matrix with vector x on the diagonal. I is the identity matrix. $\log\left(X\right)$ and $\exp\left(X\right)$ denote the elementwise natural logarithm and exponentiation of matrix X, respectively. $X^{-1}$ denotes the inverse of matrix X. The results of this table are ${\hat{\tau }}^{K}=g_{{\hat{\tau }}^{K}}$, ${\hat{\sigma }}_{E}=g_{{\hat{\sigma }}_{E}}$, ${\hat{\sigma }}_{\epsilon }=g_{{\hat{\sigma }}_{\epsilon }}$, $\partial {\hat{\tau }}^{K}/\partial n=$ $G_{{\hat{\tau }}^{K}}$, $\partial {\hat{\sigma }}_{E}/\partial n=$ $G_{{\hat{\sigma }}_{E}}$ and $\partial {\hat{\sigma }}_{\epsilon }/\partial n=$ $G_{{\hat{\sigma }}_{\epsilon }}.$

## APPENDIX F COMPUTING PARAMETERS $\tau_{\theta }$, $\sigma_{T}^{2}$, $\sigma_{X}^{2}$ And $\mu_{X}$ FROM THE DATA‐GENERATING MODEL

The data‐generating model is a two‐parameter logistic model with known item parameters ($a_{j}$ $b_{j}$; j=1,..,J) and known values for the person parameter (θ=−5,.−4.999,…,4.999,5), with a known distribution $P\left(\Theta =\theta \right)$. Let $P\left(X_{j}=1|\Theta =\theta \right)$ denote the probability of score 1 on item $X_{j}$ (j=1,…,J) given a fixed value θ, then the data‐generating model is

(F1)
$$P\left(X_{j}=1|\Theta =\theta \right)=\frac{e^{a_{j}\left(\theta -b_{j}\right)}}{1+e^{a_{j}\left(\theta -b_{j}\right)}}.$$

The true‐score for a particular θ‐value can be derived from Equation F1 by

(F2)
$$\tau_{\theta }=\sum_{j=1}^{J}P\left(X_{j}=1|\Theta =\theta \right)$$

(cf. Lord, 1980). It follows that the true‐score variance equals

(F3)
$$\sigma_{T}^{2}=\sum_{\theta }\left[{\left(\tau_{\theta }-\bar{Τ}\right)}^{2}P\left(\Theta =\theta \right)\right],$$

and the mean test score equals

(F4)
$$\mu_{X}=\mu_{T}=\sum_{\theta }\left[\tau_{\theta }P\left(\Theta =\theta \right)\right].$$

Due to the local independence assumption in the two‐parameter logistic model, the marginal and bivariate item probabilities can be derived. The marginal probabilities equal

(F5)
$$P\left(X_{j}=1\right)=\sum_{\theta }\left[P\left(X_{j}=1|\Theta =\theta \right)P\left(\Theta =\theta \right)\right],j=1,\ldots ,J$$

and the bivariate probabilities equal

(F6)
$$P\left(X_{i}=1,X_{j}=1\right)=\sum_{\theta }\left[P\left(X_{i}=1,X_{j}=1|\Theta =\theta \right)P\left(\Theta =\theta \right)\right],i,j=1,\ldots ,J;i\neq j.$$

Using these probabilities, the item variances $\sigma_{X_{j}}^{2}=P\left(X_{j}=1\right)\left[1-P\left(X_{j}=1\right)\right]$ and inter‐item covariances $\sigma_{X_{i}X_{j}}=P\left(X_{i}=1,X_{j}=1\right)-P\left(X_{i}=1\right)P\left(X_{j}=1\right)$ can be computed. Then, $\sigma_{X}^{2}$ is obtained by

(F7)
$$\sigma_{X}^{2}=\sum_{j}\sigma_{X_{j}}^{2}+2\sum_{i<j}\sigma_{X_{i}X_{j}}.$$
