# Supplementary material for: Bias and precision in true‐score estimation
Source: Br J Math Stat Psychol. 2026 Jun 15;79(3):720–43. doi: 10.1111/bmsp.70058 (PMC13679771; doi:10.1111/bmsp.70058)
Supplement: Supplementary file 1 [file BMSP-79-720-s001.docx]

# Supplementary Materials to the Paper ‘Bias and Precision in True-Score Estimation’

## Content

| **1** | **The derivation of analytic standard errors under multinomial sampling for Kelley’s true-score estimates, the standard error of measurement, and the standard error of the estimate, accompanied by R code.** | **page 1** |
| --- | --- | --- |
| 1.1 | A Two-Step Procedure | page 1 |
| 1.2 | Applications to ${\hat{\boldsymbol{\tau}}}^{\mathbf{K}}$,  ${\hat{\boldsymbol{\sigma}}}_{\boldsymbol{E}}$, and ${\hat{\boldsymbol{\sigma}}}_{\boldsymbol{\epsilon}}$ | page 4 |
| 1.3 | R code | page 9 |
| **2** | **Item Parameters in Simulation Study** | **page 15** |
| **3** | **Complete Results of the Simulation Study** | **page 18** |
| 3.1 | Terms Omitted to Simplify Bias Expressions | page 19 |
| 3.2 | Graphic Display of the Relationship Between Bias and Bias Approximation | page 22 |
| 3.3 | RMSE of the Standard Errors | page 25 |
| 3.4 | Coverage Rate of the Standard Errors | page 27 |
| 3.5 | Graphic Display of the Relationship Between Estimated and Empirical Standard Errors | Page 29 |
| **4** | Accuracy in the Classical Method and Kelley’s Method | **Page 32** |
|  | References | Page 34 |

## 1. The derivation of analytic standard errors under multinomial sampling for Kelley’s true-score estimates, the standard error of measurement, and the standard error of the estimate, accompanied by R code.

### 1.1 A Two-Step Procedure

In the two-step procedure, first, the Jacobian of a coefficient or a vector of coefficients is computed using a generalized exp-log notation, and in the second step the Delta method is applied to compute the standard errors of the coefficients.

Let $X_{1}, \ldots, X_{p}, \ldots, X_{P}$ be random variables denoting—possibly composite—scores. Let $\mathbf{r}_{p}$ be a vector of length $K_{p}$ containing the unique realizations of $X_{p}$ that were observed when the test has been administered to a simple random sample of $N$ respondents. The observed unique realizations in $\mathbf{r}_{p}$ can be real valued, but as many tests and questionnaires in the behavioral sciences are use scores that are based on $J$ dichotomous or polytomous items, the realization of $X_{p}$ will often be integer valued. The $P$ variables produce $L=\prod_{p} L_{p}$ different response patterns, which are collected in lexicographic order (i.e., last column changes fastest) in a $L\times P$ matrix $\mathbf{R}\boldsymbol{=[}\begin{matrix} \mathbf{r}_{1} & \boldsymbol{\ldots} & \mathbf{r}_{P} \end{matrix}\boldsymbol{]}$**.** Table 1 (left) shows an illustration $\mathbf{R}$ for $J=3$ items, yielding $L=8$ response patterns.

| Table 1.1:  *Example of* **R,** *the matrix with response patterns, and* $\mathbf{n}$*, the vector containing the corresponding frequencies for three dichotomous items.* | |
| --- | --- |
| $\mathbf{R}=\left[ \begin{matrix} 0 & 0 & 0 \\ 0 & 0 & 1 \\ 0 & 1 & 0 \\ 0 & 1 & 1 \\ 1 & 0 & 0 \\ 1 & 0 & 1 \\ 1 & 1 & 0 \\ 1 & 1 & 1 \end{matrix} \right]$ | $\mathbf{n}=\left[ \begin{matrix} n_{000} \\ n_{001} \\ n_{010} \\ n_{011} \\ n_{100} \\ n_{101} \\ n_{110} \\ n_{111} \end{matrix} \right]=\left[ \begin{matrix} 74 \\ 74 \\ 97 \\ 52 \\ 17 \\ 58 \\ 16 \\ 37 \end{matrix} \right]$ |

Let $\mathbf{n}$ denote the vector of observed frequencies of each row in $\mathbf{R}$**.** Table 1.1 (right) shows the vectors of observed frequencies for each of the three matrices in the upper panel with example frequencies. The subscripts of the elements of $\mathbf{n}$ describe the corresponding response pattern of the frequency. Sometimes, it is useful to use a single index $l$ ($l=1, \ldots, L$) to indicate the ordering of the elements; that is, $\mathbf{n}=\left[ n_{1}, \ldots, n_{l}, \ldots, n_{L} \right]^{T}.$Under the two-step procedure, it is assumed that the values of $\mathbf{n}$ are a random sample from a multinomial distribution $Mult\left( N, \boldsymbol{\pi}=\left[ \pi_{1}, \ldots, \pi_{l}, \ldots, \pi_{L} \right]^{T} \right),$ implying that each response pattern should have a positive probability to end up in a sample.

**Step 1. Deriving the Jacobian of coefficients using the generalized exp-log notation**.

Let $\boldsymbol{\theta}$ be a vector of population coefficients, with sample estimates $\hat{\boldsymbol{\theta}}$. If a single coefficient is of interest, $\boldsymbol{\theta}$ and $\hat{\boldsymbol{\theta}}$ reduce to scalars $\theta$ and $\hat{\theta}$, respectively. First, it must be shown that $\hat{\boldsymbol{\theta}}$ can be written as a (vector) function of $\mathbf{n}$**;** that is; $\hat{\boldsymbol{\theta}}=\mathbf{g}_{\hat{\boldsymbol{\theta}}}\left( \mathbf{n} \right)$, This facilitates the computation of the Jacobian, denoted $\mathbf{G}_{\hat{\boldsymbol{\theta}}}(\mathbf{n})$, the matrix of first partial derivatives of $\hat{\boldsymbol{\theta}}$ with respect to $\mathbf{n}$, required for deriving SEs using the delta method in Step 2. From here on, with the exception of equations 9 and 10, we leave out the argument $\mathbf{n}$ in $\mathbf{g}_{\hat{\boldsymbol{\theta}}}\left( \mathbf{n} \right)$ and $\mathbf{G}_{\hat{\boldsymbol{\theta}}}(\mathbf{n})$ to avoid lengthy notation. In general, deriving the Jacobian requires tedious derivations. The generalized exp-log notation (e.g., Kritzer, 1977; Bergsma, 1997) alleviates the burden by writing $\hat{\boldsymbol{\theta}}$ as a vector function of as a series of $Q$ appropriate design matrices, $\mathbf{A}_{1}, \mathbf{A}_{2}, \ldots, \mathbf{A}_{Q}$, such that,

$\hat{\boldsymbol{\theta}}\mathbf{=}\mathbf{g}_{\hat{\boldsymbol{\theta}}}=\mathbf{A}_{Q} .\exp\left( \mathbf{A}_{Q-1} . ln\left( \mathbf{A}_{Q-2} .\exp\left( \ldots\ln\left( \mathbf{A}_{3}. \exp\left( \mathbf{A}_{2} .\ln\left( \mathbf{A}_{1}.\mathbf{n} \right) \right) \right) \right) \right) \right),$	(1)

where $\exp(\mathbf{X})$ and $\ln(\mathbf{X})$ denote the exponential and natural logarithm, respectively, for each element of matrix $\mathbf{X}$. The generalized exp-log notation facilitates the derivation of the Jacobian because the chain rule can be applied. In some, cases functions other than $\exp(\cdot)$ and $\ln(\cdot)$ may be useful (e.g., logit, square root or difference), hence the name generalized exp-log notation. Equation 1 can be derived using the following steps.

First, a series of $Q+1$ vector functions, $\mathbf{g}_{0}$, $\mathbf{g}_{1}$, $\mathbf{g}_{2}$, …, $\mathbf{g}_{Q}$ (with the argument $\mathbf{n}$ again omitted), are defined, where $\mathbf{g}_{0}=\mathbf{n}$*,* and for $q=1, \ldots, Q-1$,

$\mathbf{g}_{q}=\ln\left( \mathbf{A}_{q}. \mathbf{g}_{q-1} \right),$ if $q$ is an odd number,	(2)

and

$\mathbf{g}_{q}=\exp\left( \mathbf{A}_{q}. \mathbf{g}_{q-1} \right),$ if $q$ is an even number.	(3)

The last function is the series $\mathbf{g}_{Q}=\mathbf{A}_{Q} ,\mathbf{g}_{Q-1}= \mathbf{g}_{\hat{\boldsymbol{\theta}}}\boldsymbol{=}\hat{\boldsymbol{\theta}}.$ Note that Equation 2 does not exist if $\mathbf{A}_{q}. \mathbf{g}_{q-1}$ contains one or more non-positive elements (see Van der Ark, 2025, for work arounds).

Next, let $\mathbf{G}_{0}$, $\mathbf{G}_{1}$, $\mathbf{G}_{2}$, …, $\mathbf{G}_{Q}$ (with the argument $\mathbf{n}$ again omitted) denote the Jacobians of $\mathbf{g}_{0}$, $\mathbf{g}_{1}$, $\mathbf{g}_{2}$, …, $\mathbf{g}_{Q}$, respectively, and let $\mathbf{I}_{p}$ denote an identity matrix of order $p$. Following standard calculus, $\mathbf{G}_{0}=\partial\mathbf{g}_{0}\backslash\partial\mathbf{n=}\mathbf{I}_{L}$. Let $\mathbf{Y}^{-1}$ denote the inverse of $\mathbf{Y}$ and let $\mathbf{D}(\mathbf{y})$ denote the diagonal matrix with vector $\mathbf{y}$ on the diagonal, then for $q=1, \ldots, Q-1$,

$\mathbf{G}_{q}=\frac{\partial\mathbf{g}_{q}}{\partial\mathbf{n}}=\mathbf{D}^{\boldsymbol{-1}} \left( \mathbf{A}_{q}. \mathbf{g}_{q-1} \right) . \mathbf{A}_{q} . \mathbf{G}_{q-1},$ if $q$ is an odd number,	(4)

and

$\mathbf{G}_{q}=\frac{\partial\mathbf{g}_{q}}{\partial\mathbf{n}}=\mathbf{D}\left( \exp\left( \mathbf{A}_{q}. \mathbf{g}_{q-1} \right) \right) . \mathbf{A}_{q} . \mathbf{G}_{q-1},$if $q$ is an even number.	(5)

The last function is the series $\mathbf{G}_{Q}=\mathbf{A}_{Q} .\mathbf{G}_{Q-1}=\mathbf{G}_{\hat{\boldsymbol{\theta}}}.$

**Step 2. Using the delta method to derive standard errors for reliability coefficients**.

If $\mathbf{n}$ is a consistent estimator, under a multinomial sampling scheme, $\mathbf{n}$converges to its true value $\boldsymbol{\nu}= N\times\boldsymbol{\pi}$, and the central limit theorem can be applied to obtain asymptotic normality,

$\left( \mathbf{n}-\boldsymbol{\nu} \right)\underset{\to}{D}N\left( \boldsymbol{0}, \mathbf{V}_{\mathbf{n}} \right),$	(6)

where $\mathbf{V}_{\mathbf{n}}$ is the variance covariance matrix of $\mathbf{n}$. Under a multinomial distribution, the sample estimate of $\mathbf{V}_{\mathbf{n}}$ equals

${\hat{\mathbf{V}}}_{\mathbf{n}}=\mathbf{D}\left( \mathbf{n} \right)-\mathbf{n}N^{-1}\mathbf{n}^{T},$	(7)

(e.g., Agresti, 2013), where, $N^{-1}$ is a $1\times1$matrix with element $1/N.$ Using the first two terms of the Taylor series,

$\mathbf{g}_{\hat{\boldsymbol{\theta}}}\left( \mathbf{n} \right)\approx\mathbf{g}_{\hat{\boldsymbol{\theta}}}\left( \boldsymbol{\nu} \right)+\mathbf{G}_{\hat{\boldsymbol{\theta}}}^{T} . \left( \mathbf{n}-\boldsymbol{\nu} \right).$	(8)

Equation 6 implies that variance of $\mathbf{g}_{\hat{\boldsymbol{\theta}}}$ equals

$\mathbf{V}_{\mathbf{g}(\mathbf{n})}\approx\mathbf{G}_{\hat{\boldsymbol{\theta}}}{\hat{\mathbf{V}}}_{\mathbf{n}}\mathbf{G}_{\hat{\boldsymbol{\theta}}}^{T}$.	(9)

Therefore,

$\left( \mathbf{g}_{\hat{\boldsymbol{\theta}}}\mathbf{(n)}-\mathbf{g}_{\hat{\boldsymbol{\theta}}}(\boldsymbol{\nu)} \right)\underset{\to}{D}N\left( \boldsymbol{0},\mathbf{G}_{\hat{\boldsymbol{\theta}}}\mathbf{V}_{\mathbf{n}}\mathbf{G}_{\hat{\boldsymbol{\theta}}}^{T} \right).$	(10)

Based on Equation 10, and using the result in Equation 7, the sample estimate of the asymptotic variance of $\mathbf{g}(\mathbf{n})$ is

${\hat{\mathbf{V}}}_{g\mathbf{(n)}}={\hat{\mathbf{V}}}_{\hat{\boldsymbol{\theta}}}=\mathbf{G}_{\hat{\boldsymbol{\theta}}}{\hat{\mathbf{V}}}_{\mathbf{n}}\mathbf{G}_{\hat{\boldsymbol{\theta}}}^{T}=\mathbf{G}_{\hat{\boldsymbol{\theta}}}\left( \mathbf{D}\left( \mathbf{n} \right)-\mathbf{n}N^{-1}\mathbf{n}^{T} \right)\mathbf{G}_{\hat{\boldsymbol{\theta}}}^{\boldsymbol{T}}\boldsymbol{=}\mathbf{G}_{\hat{\boldsymbol{\theta}}}\mathbf{D}\left( \mathbf{n} \right)\mathbf{G}_{\hat{\boldsymbol{\theta}}}^{\boldsymbol{T}}\boldsymbol{-}\mathbf{G}_{\hat{\boldsymbol{\theta}}}\mathbf{n}N^{-1}\mathbf{n}^{T}\mathbf{G}_{\hat{\boldsymbol{\theta}}}^{T}\boldsymbol{.}$	(11)

By taking the square root of the diagonal elements of ${\hat{\mathbf{V}}}_{\hat{\boldsymbol{\theta}}}$, the sample estimate of the asymptotic SE of $\hat{\boldsymbol{\theta}}$ is obtained. Equation 9 can be simplified further if $\mathbf{g}_{\hat{\boldsymbol{\theta}}}$ is homogeneous or order 0, which is true if $\mathbf{g}_{\hat{\boldsymbol{\theta}}}\left( \mathbf{n} \right)= \mathbf{g}_{\hat{\boldsymbol{\theta}}}(t\mathbf{n})$ for any positive constant $t.$ For the application here, it is useful to note that for functions homogenous of order 0, it does not matter whether observed frequencies $\mathbf{n}$ or observed probabilities $\mathbf{p} = \mathbf{n}/N$ (i.e., $t=1/N$) are used as an argument of $\mathbf{g}_{\hat{\boldsymbol{\theta}}}$. Bergsma (1997, Appendix D) showed that if $\mathbf{g}_{\hat{\boldsymbol{\theta}}}$ is homogeneous or order 0, then $\mathbf{G}_{\hat{\boldsymbol{\theta}}}\mathbf{n}N^{-1}\mathbf{n}^{T}\mathbf{G}_{\hat{\boldsymbol{\theta}}}^{T}\boldsymbol{=0},$and Equation 11 reduces to

${\hat{\mathbf{V}}}_{g\mathbf{(n)}}={\hat{\mathbf{V}}}_{\hat{\boldsymbol{\theta}}}=\mathbf{G}_{\hat{\boldsymbol{\theta}}}\mathbf{D}\left( \mathbf{n} \right)\mathbf{G}_{\hat{\boldsymbol{\theta}}}^{T}\boldsymbol{.}$	(12)

For a single coefficient (i.e., $\boldsymbol{\theta}=\theta$), $\mathbf{G}_{\hat{\boldsymbol{\theta}}}$ is a row vector of length $K$, and Equation 11 and Equation 12 can be expressed as

$\hat{V}_{\hat{\theta}}=\sum_{k} {n_{k}G}_{k}^{2}-\frac{1}{N}\sum_{k} \sum_{l} {n_{k}n_{l}G}_{k}G_{l}$	(13)

and

$\hat{V}_{\hat{\theta}}=\sum_{k} n_{k}G_{k}^{2},$	(14)

respectively. The estimated SEs, obtained by taking the square root of estimated variances (Equations 11, 12, 13, 14) typically have a rather intricate form.

### 1.2 Applications to ${\hat{\boldsymbol{\tau}}}^{\mathbf{K}}$,  ${\hat{\boldsymbol{\sigma}}}_{\boldsymbol{E}}$, and ${\hat{\boldsymbol{\sigma}}}_{\boldsymbol{\epsilon}}$

In this section design matrices $\mathbf{A}_{i}$ are introduced, for which the following equations hold:

$\mathbf{g}_{{\hat{\boldsymbol{\tau}}}^{K}}=exp\left( \mathbf{A}_{8}.ln\left( \mathbf{A}_{7}\exp\left( \mathbf{A}_{6}.ln\left( \mathbf{A}_{5}.exp\left( \mathbf{A}_{4}.ln\left( \mathbf{A}_{3}.exp\left( \mathbf{A}_{2}.ln\left( \mathbf{A}_{1}.\mathbf{n} \right) \right) \right) \right) \right) \right) \right) \right)$, (15)

$\mathbf{g}_{\hat{\sigma}_{E}}=exp\left( \mathbf{A}_{6}^{*}.ln\left( \mathbf{A}_{5}^{*}.exp\left( \mathbf{A}_{4}.ln\left( \mathbf{A}_{3}.exp\left( \mathbf{A}_{2}.ln\left( \mathbf{A}_{1}.\mathbf{n} \right) \right) \right) \right) \right) \right),$	(16)

and

$\mathbf{g}_{\hat{\sigma}_{\epsilon}}=exp\left( \mathbf{A}_{6}^{\dagger}.ln\left( \mathbf{A}_{5}^{\dagger}.exp\left( \mathbf{A}_{4}.ln\left( \mathbf{A}_{3}.exp\left( \mathbf{A}_{2}.ln\left( \mathbf{A}_{1}.\mathbf{n} \right) \right) \right) \right) \right) \right).$	(17)

#### 1.2.1 Writing Kelley’s true-score estimate ${\hat{\boldsymbol{T}}}^{\boldsymbol{K}}$ in the exp-log notation, using Cronbach's alpha as a reliability estimate.

Recall that $\mathbf{R}$ is a $J\times L$ matrix of item-score patterns in lexicographic order (Table 1); so each row is an item-score pattern. Let $\mathbf{u}_{J}$ be a vector of $J$ ones. Let $\mathbf{s}=\mathbf{u}_{J}\mathbf{R}$ be an $1\times L$ vector containing the sums of the item-score patterns in $\mathbf{R}$. Let $\mathbf{X}^{\left( 2 \right)}$ denote a matrix of the same size as $\mathbf{X}$ containing the squared elements of $\mathbf{X}$. First, the $\left( 2J+4 \right)\times L$ design matrix $\mathbf{A}_{1}$ equals

$\mathbf{A}_{1}=\left( \begin{matrix} \mathbf{R} \\ \mathbf{s} \\ \mathbf{R}^{\left( 2 \right)} \\ \mathbf{s}^{\left( 2 \right)} \\ \mathbf{u}_{J}' \\ \mathbf{u}_{J}' \end{matrix} \right)$.	(18)

For three items (scores denoted $X_{1}$, $X_{2}$, and $X_{3}$; see, Table 1), it may be verified that

$\mathbf{A}_{1}.\mathbf{n}=\left( \begin{matrix} \sum X_{1} \\ \sum X_{2} \\ \sum X_{3} \\ \sum X_{+} \\ \sum X_{1}^{2} \\ \sum X_{2}^{2} \\ \sum X_{3}^{2} \\ \sum X_{+}^{2} \\ N \\ N \end{matrix} \right)$.	(19)

Second, let $\mathbf{o}_{J}$ and $\mathbf{O}_{J}$ be a vector and matrix, respectively, containing zeroes. The $\left( 2J+5 \right)\times\left( 2J+5 \right)$ design matrix $\mathbf{A}_{2}$ equals

$\mathbf{A}_{2}=\left( \begin{matrix} 2\mathbf{I}_{J} & \mathbf{o}_{J} & \mathbf{O}_{J} & \mathbf{o}_{J} & \mathbf{o}_{J} & \mathbf{o}_{J} \\ \mathbf{o}_{J}' & 2 & \mathbf{o}_{J}' & 0 & 0 & 0 \\ \mathbf{O}_{J} & \mathbf{o}_{J} & \mathbf{I}_{J} & \mathbf{o}_{J} & \mathbf{u}_{J} & \mathbf{o}_{J} \\ \mathbf{o}_{J}' & 0 & \mathbf{o}_{J}' & 1 & 1 & 0 \\ \mathbf{o}_{J}' & 1 & \mathbf{o}_{J}' & 0 & -1 & 0 \\ \mathbf{o}_{J}' & 0 & \mathbf{o}_{J}' & 0 & 1 & 0 \\ \mathbf{o}_{J}' & 0 & \mathbf{o}_{J}' & 0 & 1 & -1 \end{matrix} \right)$.	(20)

For three items (scores denoted $X_{1}$, $X_{2}$, and $X_{3}$; see, Table 1), it may be verified that

$\exp\left( \mathbf{A}_{2}.ln\left( \mathbf{A}_{1}\mathbf{n} \right) \right)=\left( \begin{matrix} N\sum X_{1}^{2} \\ N\sum X_{2}^{2} \\ N\sum X_{3}^{2} \\ N\sum X_{+}^{2} \\ \left( \sum X_{1} \right)^{2} \\ \left( \sum X_{2} \right)^{2} \\ \left( \sum X_{3} \right)^{2} \\ \sum X_{+}^{2} \\ \overline{X} \\ N \\ 1 \end{matrix} \right)$.	(21)

Third, the $6\times\left( 2J+5 \right)$ design matrix $\mathbf{A}_{3}$ equals

$\mathbf{A}_{3}=\left( \begin{matrix} -\mathbf{u}_{J}' & 0 & \mathbf{u}_{J}' & 0 & 0 & 0 & 0 \\ \mathbf{o}_{J}' & -1 & \mathbf{o}_{J}' & 1 & 0 & 0 & 0 \\ \mathbf{o}_{J}' & 0 & \mathbf{o}_{J}' & 0 & 1 & 0 & 0 \\ \mathbf{o}_{J}' & 0 & \mathbf{o}_{J}' & 0 & 0 & 1 & 0 \\ \mathbf{o}_{J}' & 0 & \mathbf{o}_{J}' & 0 & 0 & 1 & -1 \\ \mathbf{o}_{J}' & 0 & \mathbf{o}_{J}' & 0 & 0 & 0 & 1 \end{matrix} \right)$.	(22)

Let $SS\left( X \right)=\sum_{i} \left( X_{i}-\overline{X} \right)^{2}=N\sum_{i} X_{i}-\frac{\left( \sum_{i} X_{i} \right)^{2}}{N}$ denote the sums of squares of $X$, then it may be verified that

$\mathbf{A}_{3}\exp\left( \mathbf{A}_{2}.ln\left( \mathbf{A}_{1}\mathbf{n} \right) \right)=\left( \begin{matrix} N\sum_{j} SS(X_{j}) \\ N\cdot SS(X_{+}) \\ X \\ N \\ N-1 \\ 1 \end{matrix} \right)$.	(23)

Fourth, the $4\times6$ design matrix $\mathbf{A}_{4}$ equals

$\mathbf{A}_{4}=\left( \begin{matrix} 1 & -1 & 0 & 0 & 0 & 0 \\ 0 & 1 & 0 & -1 & -1 & 0 \\ 0 & 0 & 1 & 0 & 0 & 0 \\ 0 & 0 & 0 & 0 & 0 & 1 \end{matrix} \right)$.	(24)

It may be verified that

$\begin{matrix} \exp\left( \mathbf{A}_{4}.ln\left( \mathbf{A}_{3}\exp\left( \mathbf{A}_{2}.ln\left( \mathbf{A}_{1}\mathbf{n} \right) \right) \right) \right)=\left( \begin{matrix} \frac{\sum_{j} SS\left( X_{j} \right)}{SS\left( X \right)} \\ S^{2}\left( X \right) \\ \overline{X} \\ 1 \end{matrix} \right) \end{matrix}$.	(25)

Fifth, let $K=Jm+1$ denote the number of test scores, and let $\mathbf{x}_{S}=\left( \begin{matrix} 0 & 1 & 2 & \ldots& Jm \end{matrix} \right)$. Then the $\left( K+3 \right)\times4$ design matrix $\mathbf{A}_{5}=\mathbf{A}_{5a}.\mathbf{A}_{5b}$, where

$\begin{matrix} \mathbf{A}_{5a}=\left( \begin{matrix} -\frac{J}{J-1} & 0 & 0 & \frac{J}{J-1} \\ 0 & 0 & 1 & 0 \\ \mathbf{o}_{K} & \mathbf{o}_{K} & \mathbf{o}_{K} & \mathbf{x}_{K} \end{matrix} \right) & \text{ and } & \mathbf{A}_{5b}=\left( \begin{matrix} 1 & 0 & 0 & \mathbf{0}_{K}' \\ 0 & 1 & 0 & \mathbf{0}_{K}' \\ -1 & 0 & 1 & \mathbf{0}_{K}' \\ \mathbf{o}_{K} & \mathbf{o}_{K} & \mathbf{o}_{K} & \mathbf{I}_{K} \end{matrix} \right) \end{matrix}$.	(26)

It may be verified that

$\begin{matrix} \mathbf{A}_{5a}.exp\left( \mathbf{A}_{4}.ln\left( \mathbf{A}_{3}\exp\left( \mathbf{A}_{2}.ln\left( \mathbf{A}_{1}\mathbf{n} \right) \right) \right) \right)=\left( \begin{matrix} r_{\alpha} \\ \overline{X} \\ 1 \\ \mathbf{x}_{K} \end{matrix} \right) \end{matrix}$	(27)

and

$\begin{matrix} \mathbf{A}_{5b}.\mathbf{A}_{5a}.exp\left( \mathbf{A}_{4}.ln\left( \mathbf{A}_{3}\exp\left( \mathbf{A}_{2}.ln\left( \mathbf{A}_{1}\mathbf{n} \right) \right) \right) \right)=\left( \begin{matrix} r_{\alpha} \\ \overline{X} \\ 1-r_{\alpha} \\ \mathbf{x}_{K} \end{matrix} \right) \end{matrix}$.	(28)

.

Sixth, the $\left( K+1 \right)\times\left( K+3 \right)$ design matrix $\mathbf{A}_{6}$ equals

$\mathbf{A}_{6}=\left( \begin{matrix} \mathbf{u}_{K} & \mathbf{o}_{K} & \mathbf{o}_{K} & \mathbf{I}_{K} \\ 0 & 1 & 1 & \mathbf{o}_{K}' \end{matrix} \right)$.	(29)

Let $r_{\alpha}$ denote coefficient alpha. It may be verified that

$\begin{matrix} \exp\left( \mathbf{A}_{6}.ln\left( \mathbf{A}_{5}\exp\left( \mathbf{A}_{4}.ln\left( \mathbf{A}_{3}\exp\left( \mathbf{A}_{2}.ln\left( \mathbf{A}_{1}\mathbf{n} \right) \right) \right) \right) \right) \right)=\left( \begin{matrix} r_{\alpha}\mathbf{x}_{K} \\ \left( 1-r_{\alpha} \right)\overline{X} \end{matrix} \right) \end{matrix}$.	(30)

Finally, $\mathbf{A}_{7}=\left( \mathbf{I}_{K} \mathbf{1}_{K} \right)$, and $\mathbf{A}_{8}=\mathbf{I}_{K}$. Hence the right-hand side of Equation 15 equals $\mathbf{x}_{K}r_{\alpha}+\mathbf{1}_{K}\overline{X}\left( 1-r_{\alpha} \right)={\hat{\boldsymbol{\tau}}}^{K}$.

#### 1.2.2. Writing the estimated standard error of measurement ${\hat{\boldsymbol{\sigma}}}_{\boldsymbol{E}}$ in the exp-log notation, using Cronbach's alpha as a reliability estimate.

The previous section provided design matrices $\mathbf{A}_{1}$ (Equation 18), $\mathbf{A}_{2}$ (Equation 20), $\mathbf{A}_{3}$ (Equation 22), and $\mathbf{A}_{4}$ (Equation 24). The $2\times4$ design matrix $\mathbf{A}_{5}^{*}=\mathbf{A}_{5b}^{*}.\mathbf{A}_{5a}^{*}$ where

$\begin{matrix} \mathbf{A}_{5a}^{*}=\left( \begin{matrix} -\frac{J}{J-1} & 0 & 0 & \frac{J}{J-1} \\ 0 & 1 & 0 & 0 \\ 0 & 0 & 0 & 1 \end{matrix} \right) & \text{ and } & \mathbf{A}_{5b}^{*}=\left( \begin{matrix} -1 & 0 & 1 \\ 0 & 1 & 0 \end{matrix} \right) \end{matrix}$.	(31)

It may be verified that

$\begin{matrix} \mathbf{A}_{5a}^{*}.exp\left( \mathbf{A}_{4}.ln\left( \mathbf{A}_{3}\exp\left( \mathbf{A}_{2}.ln\left( \mathbf{A}_{1}\mathbf{n} \right) \right) \right) \right)=\left( \begin{matrix} r_{\alpha} \\ S^{2}\left( X \right) \\ 1 \end{matrix} \right) \end{matrix}$	(32)

and

$\begin{matrix} \mathbf{A}_{5b}^{*}.\mathbf{A}_{5a}^{*}.exp\left( \mathbf{A}_{4}.ln\left( \mathbf{A}_{3}\exp\left( \mathbf{A}_{2}.ln\left( \mathbf{A}_{1}\mathbf{n} \right) \right) \right) \right)=\left( \begin{matrix} 1-r_{\alpha} \\ S^{2}\left( X \right) \end{matrix} \right) \end{matrix}$.	(33)

The $1\times2$ design matrix $\mathbf{A}_{6}^{*}=\left( \frac{1}{2} \frac{1}{2} \right)$, so Equation 16 equals

$\mathbf{g}_{\hat{\sigma}_{E}}\boldsymbol{=}\begin{matrix} \exp\left( \mathbf{A}_{6}^{*}.ln\left( \mathbf{A}_{5}^{*}\exp\left( \mathbf{A}_{4}.ln\left( \mathbf{A}_{3}\exp\left( \mathbf{A}_{2}.ln\left( \mathbf{A}_{1}\mathbf{n} \right) \right) \right) \right) \right) \right)=\sqrt{S_{X}^{2}(1-r_{\alpha})}= \end{matrix} \hat{\sigma}_{E}$.	(34)

#### 1.2.3. Writing the estimated standard error of estimation ${\hat{\boldsymbol{\sigma}}}_{\boldsymbol{\epsilon}}$ in the exp-log notation, using Cronbach's alpha as a reliability estimate.

Design matrices $\mathbf{A}_{1}$ (Equation 18), $\mathbf{A}_{2}$ (Equation 20), $\mathbf{A}_{3}$ (Equation 22), and $\mathbf{A}_{4}$ (Equation 24) were derived earlier. The $3\times4$ design matrix $\mathbf{A}_{5}^{\dagger}=\mathbf{A}_{5b}^{\dagger}.\mathbf{A}_{5a}^{*}$ where $\mathbf{A}_{5a}^{*}$ (Equation 31) was defined earlier, and

$\mathbf{A}_{5b}^{\dagger}=\left( \begin{matrix} 1 & 0 & 0 \\ -1 & 0 & 1 \\ 0 & 1 & 0 \end{matrix} \right)$.	(35)

Using Equation 32, it may be verified that

$\begin{matrix} \mathbf{A}_{5}^{\dagger}.exp\left( \mathbf{A}_{4}.ln\left( \mathbf{A}_{3}\exp\left( \mathbf{A}_{2}.ln\left( \mathbf{A}_{1}\mathbf{n} \right) \right) \right) \right)=\left( \begin{matrix} r_{\alpha} \\ 1-r_{\alpha} \\ S^{2}\left( X \right) \end{matrix} \right) \end{matrix}$.	(36)

The $1\times3$ design matrix $\mathbf{A}_{6}^{\dagger}=\left( \frac{1}{2} \frac{1}{2} \frac{1}{2} \right)$, so Equation 17 equals

$\mathbf{g}_{\hat{\sigma}_{\epsilon}}=\begin{matrix} \exp\left( \mathbf{A}_{6}^{\dagger}.ln\left( \mathbf{A}_{5}^{\dagger}\exp\left( \mathbf{A}_{4}.ln\left( \mathbf{A}_{3}\exp\left( \mathbf{A}_{2}.ln\left( \mathbf{A}_{1}\mathbf{n} \right) \right) \right) \right) \right) \right)=\sqrt{r_{\alpha}S^{\left( X \right)}(1-r_{\alpha}}=\hat{\sigma}_{\epsilon} \end{matrix}$.	(37)

#### 1.2.4. Deriving standard errors for ${\hat{\boldsymbol{T}}}^{\boldsymbol{K}}$,  ${\hat{\boldsymbol{\sigma}}}_{\boldsymbol{E}}$, and ${\hat{\boldsymbol{\sigma}}}_{\boldsymbol{\epsilon}}$

First, the $\mathbf{V}_{\mathbf{n}}$ should be computed from the vector of observed frequencies $\mathbf{n}$.

For $\hat{T}^{K}$, the Jacobian, $\mathbf{G}_{\hat{T}^{K}}$ should be computed using the 8 design matrices, $\mathbf{A}_{1},\ldots,\mathbf{A}_{8}$that were required to write $\hat{T}^{K}$ in the exp-log notation in equations 4 and 5. Finally, the variance covariance matrix for all true score estimates should be computed using Equation 12. The standard errors of the true score estimates are the square roots of its diagonal elements.

For$\hat{\sigma}_{E}$, the Jacobian, $G_{\hat{\sigma}_{E}}$ should be computed using design matrices, $\mathbf{A}_{1},\mathbf{A}_{\boldsymbol{2}}, \mathbf{A}_{3},\mathbf{A}_{4}, \mathbf{A}_{5}^{\mathbf{*}},\mathbf{A}_{6}^{\mathbf{*}}$that were required to write$\hat{\sigma}_{E}$ in the exp-log notation in equations 4 and 5. Finally, the variance of $\hat{\sigma}_{E}$ should be computed using Equation 14. The standard errors of the true score estimates are the square root of $\mathrm{Var}(\hat{\sigma}_{E})$.

For $\hat{\sigma}_{\epsilon}$, the Jacobian, $G_{\hat{\sigma}_{\epsilon}}$ should be computed using design matrices, $\mathbf{A}_{1},\mathbf{A}_{\boldsymbol{2}}, \mathbf{A}_{3},\mathbf{A}_{4}, \mathbf{A}_{5}^{\dagger},\mathbf{A}_{6}^{\dagger}$that were required to write$\hat{\sigma}_{\epsilon}$ in the exp-log notation in equations 4 and 5. Finally, the variance of $\hat{\sigma}_{\epsilon}$ should be computed using Equation 14. The standard errors of the true score estimates are the square root of $\mathrm{Var}(\hat{\sigma}_{\epsilon})$.

### 1.3 R code

#### 1.3.1. R code for Kelley’s true-score estimates and standard errors

```
# Compute Kelley's true score estimate and its standard error
# assuming that the reliability has been estimated using Cronbach's alpha

se_kelley <- function(X, eps = 1e-20){
  # Argument X should be an N x J matrix of integers without missing values.
  # A check is not provided
  # Argument eps is a small value: typically added to avoid computational problems
  # with logarithms of 0

  # Auxiliary functions
    # Sums of squares
      SS <- function(x) sum((x - mean(x))^2)
    # Unity matrix
      U <- function(a,b) matrix(1, a, b)
    # Matrix of zeroes
      O <- function(a,b) matrix(0, a, b)
    # Convert a (numeric) string to an intenger
      string2integer <- function(s) as.numeric(unlist(strsplit(s, NULL)))
    # Convert a data matrix to a vector of integers 1 1 1 0 0 1 becomes 111001
    #(each response pattern is a string)
      data2pattern <- function(Y){
        lab.n <- matrix(names(table(apply(Y, 1, paste, collapse = ""))))
        return(apply(lab.n, 1, string2integer))
      }
  # Miscelaneous
    # Number of items (i.e., number of columns in the data)
      J <- ncol(X)
    # R: reponse patterns R = t(X)
      R <- data2pattern(X)
    # L: Number of unique response patterns in the data
      x <- sort(unique(apply(X, 1, sum))) + eps
      L <- length(x)
    # S: Sum scores in the data
      S <- U(1, J) %*% R

  # Compute the vector of frequencies and its asymptotic covariance matrix
  # from the data
    # Compute the vector of frequencies
      n <- as.matrix(table(apply(X, 1, paste, collapse="")))
    # Compute the asymptotic covariance matrix
      Vn <- (diag(as.numeric(n)) - (n %*% t(n))/sum(n)) + eps

  # Design matrices for T.K, SEM and SEE: A1, A2, A3 and A4
    # A1
      A1 <- rbind(R, S, R^2, S^2, U(2, ncol(R)))
      A1[A1 ==0] <- eps
    # A2
      A2 <- rbind(
        cbind(diag(2, J) ,  O(J, 1 ) , O(J, J),  O(J, 1), O(J, 1), O(J, 1)),
        cbind(O(1, J)    ,  2        , O(1, J),  0      , 0      , 0),
        cbind(O(J, J)    ,  O(J, 1)  , diag(J),  O(J, 1), U(J, 1), O(J, 1)),
        cbind(O(1, J)    ,  0        , O(1, J),  1      , 1      , 0),
        cbind(O(1, J)    ,  1        , O(1, J),  0      ,-1      , 0),
        cbind(O(1, J)    ,  0        , O(1, J),  0      , 1      , 0),
        cbind(O(1, J)    ,  0        , O(1, J),  0      , 1      ,-1)
      )
    # A3
      A3 <- rbind(
        cbind(-U(1, J), 0,  U(1, J), 0, 0, 0, 0),
        cbind(O(1, J),-1,  O(1, J), 1, 0, 0, 0),
        cbind(O(1, J), 0,  O(1, J), 0, 1, 0, 0),
        cbind(O(1, J), 0,  O(1, J), 0, 0, 1, 0),
        cbind(O(1, J), 0,  O(1, J), 0, 0, 1,-1),
        cbind(O(1, J), 0,  O(1, J), 0, 0, 0, 1)
      )

    # A4
      A4 <- matrix(c(1, 0, 0, 0,
                    -1, 1, 0, 0,
                     0, 0, 1, 0,
                     0,-1, 0, 0,
                     0,-1, 0, 0,
                     0, 0, 0, 1), 4, 6)
    # A5
      A5.a <- rbind(
        matrix(c(- J / (J - 1), 0,  0, J / (J - 1),
               0              , 0,  1, 0,
               0              , 0,  0, 1), 3, 4, byrow = TRUE),
          cbind(O(L, 1), O(L, 1), O(L, 1), x)
         )
      A5.b <- rbind(
          cbind(U(1, 1), O(1, 1), O(1, 1), O(1, L)),
          cbind(O(1, 1), U(1, 1), O(1, 1), O(1, L)),
          cbind(-U(1, 1),O(1, 1), U(1, 1), O(1, L)),
          cbind(O(L, 1), O(L, 1), O(L, 1), diag(L))
      )
      A5 <- A5.b %*% A5.a
    # A6
      A6 <- rbind(
          cbind(U(L, 1), O(L, 1), O(L, 1), diag(L)),
          cbind(O(1, 1), U(1, 1), U(1, 1), O(1, L))
      )

    # A7
      A7 <- cbind(diag(L), U(L, 1))
    # A8 (could be removed)
      A8 <- diag(L)

  # Computation of T.K using the exp-log notation
      g0 <- n
      g1 <- log(A1 %*% g0)
      g2 <- exp(A2 %*% g1)
      g3 <- log(A3 %*% g2)
      g4 <- exp(A4 %*% g3)
      g5 <- log(A5 %*% g4)
      g6 <- exp(A6 %*% g5)
      g7 <- log(A7 %*% g6)
      T.K <- g8 <- exp(A8 %*% g7)

  # Computation of the Jacobian of T.K and the standard errors
      G0 <- diag(length(n))
      G1 <- diag(as.numeric(1 / (A1 %*% g0))) %*% A1 %*% G0
      G2 <- diag(as.numeric(exp(A2 %*% g1)))  %*% A2 %*% G1
      G3 <- diag(as.numeric(1 / (A3 %*% g2))) %*% A3 %*% G2
      G4 <- diag(as.numeric(exp(A4 %*% g3)))  %*% A4 %*% G3
      G5 <- diag(as.numeric(1 / (A5 %*% g4))) %*% A5 %*% G4
      G6 <- diag(as.numeric(exp(A6 %*% g5)))  %*% A6 %*% G5
      G7 <- diag(as.numeric(1 / (A7 %*% g6))) %*% A7 %*% G6
      G8 <- diag(as.numeric(exp(A8 %*% g7)))  %*% A8 %*% G7
      se.T.K <- sqrt(diag(G8 %*% Vn %*% t(G8)))
      se.T.K[!is.na(se.T.K) & se.T.K > J] <- NA

  # output list containing (1) unique observed sum scores, (2) Corresponding Kelley's
  # true-score estimates, and (3)corresponding standard errors
     output  <- matrix(c(round(x, 4), T.K, se.T.K), nrow = L, ncol = )
     dimnames(output)[[2]] <- c("Classic estim", "Kelley estim", "SE Kelley estim")
     return(output)
}
```

#### 1.3.2. R code for the SEM and its standard error

```
# Compute estimated SEM and its standard error
# assuming that the reliability has been estimated using Cronbach's alpha

se_sem <- function(X, eps = 1e-20){
  # Argument X should be an N x J matrix of integers without missing values.
  # A check is not provided
  # Argument eps is a small value: typically added to avoid computational problems
  # with logarithms of 0

  # Auxiliary functions
    # Sums of squares
      SS <- function(x) sum((x - mean(x))^2)
    # Unity matrix
      U <- function(a,b) matrix(1, a, b)
    # Matrix of zeroes
      O <- function(a,b) matrix(0, a, b)
    # Convert a (numeric) string to an intenger
      string2integer <- function(s) as.numeric(unlist(strsplit(s, NULL)))
    # Convert a data matrix to a vector of integers 1 1 1 0 0 1 becomes 111001
    #(each response pattern is a string)
      data2pattern <- function(Y){
        lab.n <- matrix(names(table(apply(Y, 1, paste, collapse = ""))))
        return(apply(lab.n, 1, string2integer))
      }
  # Miscelaneous
    # Number of items (i.e., number of columns in the data)
      J <- ncol(X)
    # R: reponse patterns R = t(X)
      R <- data2pattern(X)
    # L: Number of unique response patterns in the data
      x <- sort(unique(apply(X, 1, sum))) + eps
      L <- length(x)
    # S: Sum scores in the data
      S <- U(1, J) %*% R

  # Compute the vector of frequencies and its asymptotic covariance matrix
  # from the data
    # Compute the vector of frequencies
      n <- as.matrix(table(apply(X, 1, paste, collapse="")))
    # Compute the asymptotic covariance matrix
      Vn <- (diag(as.numeric(n)) - (n %*% t(n))/sum(n)) + eps

  # Design matrices for T.K, SEM and SEE: A1, A2, A3 and A4
    # A1
      A1 <- rbind(R, S, R^2, S^2, U(2, ncol(R)))
      A1[A1 ==0] <- eps
    # A2
      A2 <- rbind(
        cbind(diag(2, J) ,  O(J, 1 ) , O(J, J),  O(J, 1), O(J, 1), O(J, 1)),
        cbind(O(1, J)    ,  2        , O(1, J),  0      , 0      , 0),
        cbind(O(J, J)    ,  O(J, 1)  , diag(J),  O(J, 1), U(J, 1), O(J, 1)),
        cbind(O(1, J)    ,  0        , O(1, J),  1      , 1      , 0),
        cbind(O(1, J)    ,  1        , O(1, J),  0      ,-1      , 0),
        cbind(O(1, J)    ,  0        , O(1, J),  0      , 1      , 0),
        cbind(O(1, J)    ,  0        , O(1, J),  0      , 1      ,-1)
      )
    # A3
      A3 <- rbind(
        cbind(-U(1, J), 0,  U(1, J), 0, 0, 0, 0),
        cbind(O(1, J),-1,  O(1, J), 1, 0, 0, 0),
        cbind(O(1, J), 0,  O(1, J), 0, 1, 0, 0),
        cbind(O(1, J), 0,  O(1, J), 0, 0, 1, 0),
        cbind(O(1, J), 0,  O(1, J), 0, 0, 1,-1),
        cbind(O(1, J), 0,  O(1, J), 0, 0, 0, 1)
      )

    # A4
      A4 <- matrix(c(1, 0, 0, 0,
                    -1, 1, 0, 0,
                     0, 0, 1, 0,
                     0,-1, 0, 0,
                     0,-1, 0, 0,
                     0, 0, 0, 1), 4, 6)
      # A5.
      A5.a. <- matrix(c(- J / (J - 1), 0,  0, J / (J - 1),
                       0              , 1,  0, 0,
                       0              , 0,  0, 1), 3, 4, byrow = TRUE)
      A5.b. <- matrix(c(- 1, 0,  1,
                          0, 1,  0), 2, 3, byrow = TRUE)
      A5. <- A5.b. %*% A5.a.

    # A6.
      A6. <- matrix(c(.5, .5), 1, 2)

  # Computation of SEM using the exp-log notation
      g0 <- n
      g1 <- log(A1 %*% g0)
      g2 <- exp(A2 %*% g1)
      g3 <- log(A3 %*% g2)
      g4 <- exp(A4 %*% g3)
      g5. <- log(A5. %*% g4)
      sem <- g6. <- exp(A6. %*% g5.)
      if (!is.na(sem) & sem > J) sem <- NA

  # Computation of the Jacobian of the estimated SEM and the standard error
      G0 <- diag(length(n))
      G1 <- diag(as.numeric(1 / (A1 %*% g0))) %*% A1 %*% G0
      G2 <- diag(as.numeric(exp(A2 %*% g1)))  %*% A2 %*% G1
      G3 <- diag(as.numeric(1 / (A3 %*% g2))) %*% A3 %*% G2
      G4 <- diag(as.numeric(exp(A4 %*% g3)))  %*% A4 %*% G3
      G5. <- diag(as.numeric(1 / (A5. %*% g4))) %*% A5. %*% G4
      G6. <- exp(A6. %*% g5.)  %*% A6. %*% G5.
      se.sem <- sqrt(diag(G6. %*% Vn %*% t(G6.)))
      if (!is.na(se.sem) & se.sem > J) se.sem <- NA

  # output list containing (1) estimated SEM,and (2)corresponding standard error
     output  <- matrix(c(sem, se.sem), nrow = 1, ncol = 2)
     dimnames(output)[[2]] <- c("sem", "SE(sem)")
     return(output)
}
```

#### 1.3.3. R code for the SEE and its standard error

```
# Compute estimated SEE and its standard error
# assuming that the reliability has been estimated using Cronbach's alpha

se_see <- function(X, eps = 1e-20){
  # Argument X should be an N x J matrix of integers without missing values.
  # A check is not provided
  # Argument eps is a small value: typically added to avoid computational problems
  # with logarithms of 0

  # Auxiliary functions
    # Sums of squares
      SS <- function(x) sum((x - mean(x))^2)
    # Unity matrix
      U <- function(a,b) matrix(1, a, b)
    # Matrix of zeroes
      O <- function(a,b) matrix(0, a, b)
    # Convert a (numeric) string to an intenger
      string2integer <- function(s) as.numeric(unlist(strsplit(s, NULL)))
    # Convert a data matrix to a vector of integers 1 1 1 0 0 1 becomes 111001
    #(each response pattern is a string)
      data2pattern <- function(Y){
        lab.n <- matrix(names(table(apply(Y, 1, paste, collapse = ""))))
        return(apply(lab.n, 1, string2integer))
      }
  # Miscelaneous
    # Number of items (i.e., number of columns in the data)
      J <- ncol(X)
    # R: reponse patterns R = t(X)
      R <- data2pattern(X)
    # L: Number of unique response patterns in the data
      x <- sort(unique(apply(X, 1, sum))) + eps
      L <- length(x)
    # S: Sum scores in the data
      S <- U(1, J) %*% R

  # Compute the vector of frequencies and its asymptotic covariance matrix
  # from the data
    # Compute the vector of frequencies
      n <- as.matrix(table(apply(X, 1, paste, collapse="")))
    # Compute the asymptotic covariance matrix
      Vn <- (diag(as.numeric(n)) - (n %*% t(n))/sum(n)) + eps

  # Design matrices for T.K, SEM and SEE: A1, A2, A3 and A4
    # A1
      A1 <- rbind(R, S, R^2, S^2, U(2, ncol(R)))
      A1[A1 ==0] <- eps
    # A2
      A2 <- rbind(
        cbind(diag(2, J) ,  O(J, 1 ) , O(J, J),  O(J, 1), O(J, 1), O(J, 1)),
        cbind(O(1, J)    ,  2        , O(1, J),  0      , 0      , 0),
        cbind(O(J, J)    ,  O(J, 1)  , diag(J),  O(J, 1), U(J, 1), O(J, 1)),
        cbind(O(1, J)    ,  0        , O(1, J),  1      , 1      , 0),
        cbind(O(1, J)    ,  1        , O(1, J),  0      ,-1      , 0),
        cbind(O(1, J)    ,  0        , O(1, J),  0      , 1      , 0),
        cbind(O(1, J)    ,  0        , O(1, J),  0      , 1      ,-1)
      )
    # A3
      A3 <- rbind(
        cbind(-U(1, J), 0,  U(1, J), 0, 0, 0, 0),
        cbind(O(1, J),-1,  O(1, J), 1, 0, 0, 0),
        cbind(O(1, J), 0,  O(1, J), 0, 1, 0, 0),
        cbind(O(1, J), 0,  O(1, J), 0, 0, 1, 0),
        cbind(O(1, J), 0,  O(1, J), 0, 0, 1,-1),
        cbind(O(1, J), 0,  O(1, J), 0, 0, 0, 1)
      )

    # A4
      A4 <- matrix(c(1, 0, 0, 0,
                    -1, 1, 0, 0,
                     0, 0, 1, 0,
                     0,-1, 0, 0,
                     0,-1, 0, 0,
                     0, 0, 0, 1), 4, 6)
      # A5..
      A5.a. <- matrix(c(- J / (J - 1), 0,  0, J / (J - 1),
                       0              , 1,  0, 0,
                       0              , 0,  0, 1), 3, 4, byrow = TRUE)
      A5.b.. <- matrix(c(   1, 0,  0,
                    - 1, 0,  1,
                   0, 1,  0), 3, 3, byrow = TRUE)
      A5.. <- A5.b.. %*% A5.a.

     # A6..
     A6.. <- matrix( .5, 1, 3)

  # Computation of SEE using the exp-log notation
      g0 <- n
      g1 <- log(A1 %*% g0)
      g2 <- exp(A2 %*% g1)
      g3 <- log(A3 %*% g2)
      g4 <- exp(A4 %*% g3)
      g5.. <- log(A5.. %*% g4)
      see <- g6.. <- exp(A6.. %*% g5..)
      if (!is.na(see) & see > J) see <- NA

  # Computation of the Jacobian of estimated SEE and its standard error
      G0 <- diag(length(n))
      G1 <- diag(as.numeric(1 / (A1 %*% g0))) %*% A1 %*% G0
      G2 <- diag(as.numeric(exp(A2 %*% g1)))  %*% A2 %*% G1
      G3 <- diag(as.numeric(1 / (A3 %*% g2))) %*% A3 %*% G2
      G4 <- diag(as.numeric(exp(A4 %*% g3)))  %*% A4 %*% G3
      G5.. <- diag(as.numeric(1 / (A5.. %*% g4))) %*% A5.. %*% G4
      G6.. <- exp(A6.. %*% g5..)  %*% A6.. %*% G5..
      se.see <- sqrt(diag(G6.. %*% Vn %*% t(G6..)))
      if (!is.na(se.see) & se.see > J) se.see <- NA

  # output list containing (1) estimated SEE and(2)corresponding standard error
     output  <- matrix(c(see, se.see), nrow = 1, ncol = 2)
     dimnames(output)[[2]] <- c("see", "SE(see)")
     return(output)
}
```

#### 1.3.4. Example

```
# library 'mokken' should be installed to run this example

# load package and get data
library(mokken)
data(acl)

# Get item scores (0-4) on four scales
com <- acl[,  1:10] # Communality scale (10 items)
ach <- acl[, 11:20] # Achievement scale (10 items)
dom <- acl[, 21:30] # Dominance scale (10 items)
end <- acl[, 31:40] # Endurance scale (10 items)

# COMMUNALITY
se_kelley(com)
se_sem(com)
se_see(com)

# ACHIEVEMENT
se_kelley(ach)
se_sem(ach)
se_see(ach)

# DOMINANCE
se_kelley(com)
se_sem(dom)
se_see(dom)

# ENDURANCE
se_kelley(end)
se_sem(end)
se_see(end)
```

## 2. Item parameters in Simulation Study

The table below contains the discrimination parameters (a) and the difficulty parameters (b) that were used in the simulation studies. The item parameters are presented in the IRT format (i.e., not in the regression format) and without the constant 1.72. Parameters are presented for four levels of reliability ($\rho_{X}$), four levels of biased of the reliability estimator Cronbach’s alpha (Bias) and two levels of numbers of items (items). It should be noted to obtain unbiased reliability estimates using Cronbach’s alpha the discrimination parameters and location parameters must be the same for all items.

| $\rho_{X}$ | Bias | Items | |
| --- | --- | --- | --- |
|  |  | 6 | 12 |
| 0.6 | 0 | a d  X1 1.106692 0  X2 1.106692 0  X3 1.106692 0  X4 1.106692 0  X5 1.106692 0  X6 1.106692 0 | a d  X1 0.7475778 0  X2 0.7475778 0  X3 0.7475778 0  X4 0.7475778 0  X5 0.7475778 0  X6 0.7475778 0  X7 0.7475778 0  X8 0.7475778 0  X9 0.7475778 0  X10 0.7475778 0  X11 0.7475778 0  X12 0.7475778 0 |
| 0.6 | -0.02 | a d  X1 0.5045519 -0.46010154  X2 1.5099734 0.03537474  X3 1.6088735 -1.41180359  X4 1.5843320 0.33445468  X5 0.8384850 -1.69297091  X6 1.6509589 0.45259331 | a d  X1 1.7315064 -2.36066209  X2 1.0215350 1.23107627  X3 0.7351177 -0.25207031  X4 0.7150670 -0.46727746  X5 0.2390302 2.46714935  X6 1.2842756 0.54116770  X7 0.2861038 -2.33796733  X8 1.9496496 0.07156663  X9 0.4632856 -1.40515345  X10 1.1829587 0.62914886  X11 1.7619211 2.90618637  X12 1.0356437 -0.93353384 |
| 0.6 | -0.05 | a d  X1 0.726577 -1.3117250  X2 1.452164 -2.2828486  X3 2.350854 -1.6676287  X4 1.723977 -1.7166966  X5 2.101104 -2.9528081  X6 2.338230 -0.5486061 | a d  X1 2.1217039 -1.93283816  X2 0.7322702 -2.46035525  X3 0.9365498 -2.72325115  X4 2.8063132 0.02163373  X5 2.2228918 1.88378026  X6 3.6355630 1.80507821  X7 0.6141454 -0.19471654  X8 0.9080250 2.19103692  X9 3.4696253 -2.73398769  X10 0.9152349 -2.53328097  X11 3.5820943 2.19139733  X12 0.3613778 2.42664089 |
| 0.6 | -0.10 | a d  X1 2.8323097 -0.8835335  X2 0.1327929 0.8352823  X3 2.1810477 1.2285994  X4 2.4489536 1.6409864  X5 3.8314199 -2.3536979  X6 3.3663241 0.9848478 | a d  X1 5.1468185 -2.0260639  X2 3.7046669 2.4359922  X3 3.4738250 -2.8847269  X4 4.0422137 -2.1397365  X5 5.2503319 2.5457309  X6 4.5424182 1.4748475  X7 0.2375611 -2.9194466  X8 4.8862285 0.7502846  X9 0.3361452 1.5983753  X10 5.0593320 -1.9996606  X11 0.9216219 2.9274731  X12 2.0818910 2.4219810 |
| 0.7 | 0 | a d  X1 1.442078 0  X2 1.442078 0  X3 1.442078 0  X4 1.442078 0  X5 1.442078 0  X6 1.442078 0 | a d  X1 0.9572295 0  X2 0.9572295 0  X3 0.9572295 0  X4 0.9572295 0  X5 0.9572295 0  X6 0.9572295 0  X7 0.9572295 0  X8 0.9572295 0  X9 0.9572295 0  X10 0.9572295 0  X11 0.9572295 0  X12 0.9572295 0 |

| $\rho_{X}$ | Bias | Items | |
| --- | --- | --- | --- |
|  |  | 6 | 12 |
| 0.7 | -0.02 | a d  X1 1.6922434 1.8143224  X2 1.5558583 1.3853506  X3 1.3044396 0.1616530  X4 0.9676599 1.0365033  X5 2.3307644 0.1707703  X6 2.3863662 0.5516869 | a d  X1 1.3147104 -0.4355885  X2 2.3248313 1.4420256  X3 2.4432213 -0.2189444  X4 0.7069078 -1.1057644  X5 0.4421089 -2.6223951  X6 1.1191938 0.5824922  X7 1.7873701 0.6320205  X8 1.0857565 1.3204879  X9 1.6853031 2.8300235  X10 1.8038525 1.6321421  X11 0.7183968 0.3649806  X12 0.4565176 1.4611385 |
| 0.7 | -0.05 | a d  X1 2.5894503 0.6856240  X2 2.7808227 1.1098294  X3 3.8608030 1.2217882  X4 0.1461687 -2.2762247  X5 1.2532803 0.5733856  X6 2.9614430 -0.3701312 | a d  X1 1.1557742 0.4142348  X2 2.2454104 1.6422868  X3 1.8085620 -1.2619220  X4 1.6431983 -2.7579600  X5 0.3120946 -1.1184949  X6 1.8109834 1.9582364  X7 2.3755707 2.5651018  X8 3.2406935 -1.1522171  X9 2.3825014 2.7241487  X10 1.2462769 -1.4277863  X11 3.4696015 1.0683623  X12 2.3996291 2.1012495 |
| 0.7 | -0.10 | a d  X1 1.200017 -1.6799786  X2 2.981606 0.8842389  X3 3.173180 2.7937121  X4 1.657088 2.2271510  X5 2.529675 -0.1046592  X6 2.819377 -1.1341164 | a d  X1 6.0233119 -2.7519682  X2 0.4604089 -2.8818778  X3 0.7552083 0.8431627  X4 4.1535654 2.2170092  X5 2.3103574 -2.2003521  X6 2.4902010 2.5794366  X7 4.8507602 2.0266498  X8 6.2162845 1.8466295  X9 5.0640154 2.0671599  X10 1.4655468 2.5806539  X11 4.9447404 0.1399657  X12 4.0765276 2.3988708 |
| 0.8 | 0 | a d  X1 2.031809 0  X2 2.031809 0  X3 2.031809 0  X4 2.031809 0  X5 2.031809 0  X6 2.031809 0 | a d  X1 1.312867 0  X2 1.312867 0  X3 1.312867 0  X4 1.312867 0  X5 1.312867 0  X6 1.312867 0  X7 1.312867 0  X8 1.312867 0  X9 1.312867 0  X10 1.312867 0  X11 1.312867 0  X12 1.312867 0 |
| 0.8 | -0.02 | a d  X1 4.789984 0.1700867  X2 3.565450 0.8963537  X3 3.746844 0.9934558  X4 1.220811 0.2251660  X5 1.386196 0.2989127  X6 1.809576 0.8266085 | a d  X1 2.2922285 0.03976300  X2 2.7329901 -2.05040531  X3 0.5603145 1.51853002  X4 1.6688383 0.06118701  X5 1.8151955 1.53718536  X6 0.6252843 1.47865844  X7 2.5945033 -0.07294261  X8 1.1574023 -0.13971547  X9 2.1853568 0.27973365  X10 1.1343243 -0.83429164  X11 2.4626938 0.44294374  X12 1.2415220 1.96137798 |

| $\rho_{X}$ | Bias | Items | |
| --- | --- | --- | --- |
|  |  | 6 | 12 |
| 0.8 | -0.05 | a d  X1 2.5543234 1.8474566  X2 0.9066841 1.3514496  X3 3.4858844 -1.0138951  X4 2.2254552 -0.6553384  X5 3.4446344 -0.4907956  X6 4.6246597 -0.4139000 | a d  X1 1.5087804 -1.6243562  X2 3.8561319 -1.7603177  X3 3.5109856 0.1447548  X4 3.4618582 1.7201893  X5 0.9442161 -2.4919700  X6 2.3558050 0.1885176  X7 2.9083503 -0.8938609  X8 3.1911348 1.9463598  X9 3.9072936 -0.2375985  X10 0.6251528 -1.7692058  X11 0.9521520 2.0854533  X12 0.5699891 -2.6001883 |
| 0.8 | -0.10 | a d  X1 6.361243 1.7632380  X2 6.334761 -1.5081250  X3 5.780428 -0.1457378  X4 5.891047 -0.6262810  X5 1.244207 -1.8645796  X6 1.177987 -0.7461322 | a d  X1 4.1744190 0.1914561  X2 4.5289022 1.8909683  X3 3.2835717 -1.8052675  X4 3.5637810 2.3898917  X5 3.2232492 1.2002064  X6 1.1867800 -2.9036636  X7 0.8131502 -0.3128499  X8 4.0480025 0.3633656  X9 3.1700091 -1.8418959  X10 4.6506338 -2.5336578  X11 3.3327253 2.4852965 |
| 0.9 | 0 | a d  X1 3.578001 0  X2 3.578001 0  X3 3.578001 0  X4 3.578001 0  X5 3.578001 0  X6 3.578001 0 | a d  X1 2.197161 0  X2 2.197161 0  X3 2.197161 0  X4 2.197161 0  X5 2.197161 0  X6 2.197161 0  X7 2.197161 0  X8 2.197161 0  X9 2.197161 0  X10 2.197161 0  X11 2.197161 0  X12 2.197161 0 |
| 0.9 | -0.02 | a d  X1 7.378531 -0.04713531  X2 1.708221 0.25112429  X3 4.450913 -0.34268989  X4 4.726856 -0.09594802  X5 5.913651 -0.64823909  X6 4.161956 -1.06893737 | a d  X1 3.5191543 -0.40247809  X2 1.8726608 0.13544109  X3 3.9334585 -0.04105616  X4 0.9249944 -1.54771376  X5 2.7605024 -0.80598356  X6 1.4357531 -1.98799383  X7 3.4804001 -1.09568703  X8 3.9229138 -0.99624067  X9 3.1586277 -0.24281210  X10 2.9531015 -0.42848007  X11 1.7502984 -2.83547625  X12 3.6834492 -0.78454603 |
| 0.9 | -0.05 | a d  X1 10.515223 1.0331320  X2 3.098215 0.8748026  X3 4.442139 1.3735785  X4 2.315763 2.7339832  X5 6.657963 1.4306803  X6 5.925635 0.8240479 | a d  X1 1.622439 0.1892750  X2 6.877858 0.7834767  X3 2.927876 -2.4761033  X4 5.627647 0.5039715  X5 3.157614 -1.7366333  X6 3.806472 2.4787114  X7 4.725212 0.9772498  X8 5.662445 1.2878171  X9 2.020268 1.8140794  X10 3.712448 -0.9073332  X11 5.219200 0.8294169  X12 1.954604 0.4589875 |

| $\rho_{X}$ | Bias | Items | |
| --- | --- | --- | --- |
|  |  | 6 | 12 |
| 0.9 | -0.10 | a d  X1 2.968143 -0.5883914  X2 5.401549 0.6845102  X3 18.431634 -0.7504120  X4 5.497849 0.1331292  X5 4.999418 1.0638257  X6 6.938417 -1.5869806 | a d  X1 7.506854 1.9033644  X2 7.798861 -1.0221654  X3 2.761012 -1.0758142  X4 4.793775 -1.3478342  X5 3.688862 1.4340158  X6 4.662787 -1.0471988  X7 3.623635 2.4485626  X8 8.537923 -2.1888002  X9 6.962969 -2.1575914  X10 5.242656 0.3316412  X11 7.069197 -1.8435023  X12 3.536066 1.1999493 |

## 3. Complete Results of the Simulation Study

The complete results in the simulation study are both tables and figures.

Tables show the numeric values for all 96 conditions in the simulation study and are presented in $16\times6$ tables, with the levels of reliability and bias of the reliability (both have 4 levels) in the rows and number of items (2 levels) and sample size (3 levels) in the columns.

Figures show relationships between two quantities for all 96 conditions, where the different levels of reliability, bias of the reliability, and sample size are indicated by the shape, color and size, respectively, of the symbol in the figure. Number of items cannot be distinguished.

### 3.1 Terms Omitted to Simplify Bias Expressions

Table 2 in the paper provides the summary statistics of the distributions of the terms omitted to simplify the bias expressions, across the 96 study conditions. The tables below provide the complete results (multiplied by 10,000 to facilitate readability for:

$\mathrm{Cov}\left( \hat{\rho}_{X}, X_{l} \right)$

```
            J= 6 N= 200 J= 6 N= 500 J= 6 N=2000 J=12 N= 200 J=12 N= 500 J=12 N=2000
R=.6 B= .00         -18         -36           0         -12          -2          -1
R=.6 B=-.02          -1          -9          -8          -3          -2         -12
R=.6 B=-.05          -1         -30         -14          -1          -2          -1
R=.6 B=-.10         -45          -1          -1          -7          -2         -20
R=.7 B= .00          -9          -2        -104          -8           0           0
R=.7 B=-.02          -1         -50          -2          -4          -6          -8
R=.7 B=-.05          -7          -9           0         -27         -12           0
R=.7 B=-.10          -1          -3         -47          -1          -1          -2
R=.8 B= .00         -22         -12          -1           0         -12         -27
R=.8 B=-.02         -15          -2           0         -48           0          -7
R=.8 B=-.05           0           0         -42          -4           0          -2
R=.8 B=-.10         -27         -24          -3          -2         -28          -3
R=.9 B= .00          -1          -3          -1          -4          -2           0
R=.9 B=-.02          -8           0         -23          -1           0         -23
R=.9 B=-.05          -7          -1           2           0          -5          -8
R=.9 B=-.10          -2         -19         -10         -27          -1           0
```

$\mathrm{Cov}\left( \hat{\rho}_{X}, X_{m} \right)$

```
            J= 6 N= 200 J= 6 N= 500 J= 6 N=2000 J=12 N= 200 J=12 N= 500 J=12 N=2000
R=.6 B= .00           4          -1          -1           0           1          -1
R=.6 B=-.02          -2          -5          -1          -1          -4           0
R=.6 B=-.05           0          -7           7           3           0           1
R=.6 B=-.10           1          -3           2           0          -8           1
R=.7 B= .00          -6           0          13          -5           0           1
R=.7 B=-.02           1          -4           2           1           4           9
R=.7 B=-.05           5           3           0           2          -7           0
R=.7 B=-.10          -1           0          -1          -2          -3           2
R=.8 B= .00          12           2           2           0          -1           7
R=.8 B=-.02           5           1           1          -9          -3           0
R=.8 B=-.05           5           0           2           0           0          -6
R=.8 B=-.10          -7          -3          -1           1          -5           5
R=.9 B= .00           2           3           3          -1           0           0
R=.9 B=-.02          -4          -4           3           2           0          -4
R=.9 B=-.05           3           2          -3          -1           1           3
R=.9 B=-.10           3          -1           3          -4          -1           0
```

$\mathrm{Cov}\left( \hat{\rho}_{X}, X_{h} \right)$

```
            J= 6 N= 200 J= 6 N= 500 J= 6 N=2000 J=12 N= 200 J=12 N= 500 J=12 N=2000
R=.6 B= .00          24          51           0           0           1           1
R=.6 B=-.02          15           8           5           1          11          13
R=.6 B=-.05           0          17          98           0           6           3
R=.6 B=-.10          56          10           0           5          11           7
R=.7 B= .00          12           0           4           6           4           1
R=.7 B=-.02           0          36           6           1           4          12
R=.7 B=-.05          12          20           0          46          15           0
R=.7 B=-.10           4           3          76           1           3           1
R=.8 B= .00           6          10          53           0           6          11
R=.8 B=-.02          27           2           0          85           3           2
R=.8 B=-.05           7           0           4           5           1          37
R=.8 B=-.10          20          14           1           0          27           4
R=.9 B= .00           3           5           1          27           5           0
R=.9 B=-.02          28           0          25           0           0          45
R=.9 B=-.05           1           2          15          12           7           4
R=.9 B=-.10           3          13           1          22           1           0
```

$\mathrm{Cov}\left( \hat{\rho}_{X}, \hat{\mu}_{X} \right)$

```
            J= 6 N= 200 J= 6 N= 500 J= 6 N=2000 J=12 N= 200 J=12 N= 500 J=12 N=2000
R=.6 B= .00           0          10           0          -2           0          -1
R=.6 B=-.02           3           1           0           0          11           0
R=.6 B=-.05          -1          -2          35          -2           1           0
R=.6 B=-.10          -2           5           0           1           9         -10
R=.7 B= .00           4          -1         -17           1           4           0
R=.7 B=-.02           0           1           0          -3           1          -6
R=.7 B=-.05           0           4           0           9           3           0
R=.7 B=-.10           1           0           6           0           4           0
R=.8 B= .00          -8           0          15           0           0          -2
R=.8 B=-.02           0           1           0           9           4          -4
R=.8 B=-.05           1           0          -6           0           1           8
R=.8 B=-.10           1           1           0          -1          -1          -3
R=.9 B= .00           0           1          -4           4           1           0
R=.9 B=-.02          11           0           3           0           1          11
R=.9 B=-.05          -3           0           4           9           0          -1
R=.9 B=-.10           0           0          -8           4           1          -1
```

$\mathrm{Cov}\left( \hat{\sigma}_{X}^{2},\hat{\rho}_{X} \right)$

```
            J= 6 N= 200 J= 6 N= 500 J= 6 N=2000 J=12 N= 200 J=12 N= 500 J=12 N=2000
R=.6 B= .00          95         140          12          10          16          33
R=.6 B=-.02          30          37          17          56          89          81
R=.6 B=-.05           5          82         250          14           7          12
R=.6 B=-.10         225          34          39          14          67         102
R=.7 B= .00          67           5          97         151          23           7
R=.7 B=-.02          10         172          17          39          15          47
R=.7 B=-.05          37          55           3          69         120          14
R=.7 B=-.10           7          10         143          22          36          20
R=.8 B= .00          53          33         110           4          75          99
R=.8 B=-.02          92           8           9         152          26          40
R=.8 B=-.05          16          30          36          61           6          75
R=.8 B=-.10          90          74           4          10         192          18
R=.9 B= .00           9          14          35          30          49           3
R=.9 B=-.02          82          93          56           6           9         166
R=.9 B=-.05          21           8          26          57          30          41
R=.9 B=-.10          22          40          97          65           7          10
```

$1/8\sigma_{E}^{3}\mathrm{Var}\left( \hat{\sigma}_{E}^{2} \right)$

```
            J= 6 N= 200 J= 6 N= 500 J= 6 N=2000 J=12 N= 200 J=12 N= 500 J=12 N=2000
R=.6 B= .00           2           2           3           1           0           1
R=.6 B=-.02           1           1           0           4           2           0
R=.6 B=-.05           0           2           3           2           0           0
R=.6 B=-.10           0           1           1           0           4           6
R=.7 B= .00           0           0           6           1           8           0
R=.7 B=-.02           0           1           1           1           0           3
R=.7 B=-.05           1           1           1           3           1           0
R=.7 B=-.10           0           0           1           2           1           0
R=.8 B= .00           4           1           1           1           2           1
R=.8 B=-.02           0           0           0           4           2           3
R=.8 B=-.05           0           9           2           0           2           3
R=.8 B=-.10           2           0           0           0           1           1
R=.9 B= .00           0           0           5           1           0           0
R=.9 B=-.02           2           3           1           0           0           1
R=.9 B=-.05           2           0           0          20           1           0
R=.9 B=-.10           0           2           3           2           0           1
```

$\mathrm{Cov}\left( \hat{\sigma}_{X}^{2},\hat{\rho}_{X}-\hat{\rho}_{X}^{2} \right)$

```
            J= 6 N= 200 J= 6 N= 500 J= 6 N=2000 J=12 N= 200 J=12 N= 500 J=12 N=2000
R=.6 B= .00         -19         -41         -10          -1          -9         -20
R=.6 B=-.02         -11         -15          -3         -33         -62         -32
R=.6 B=-.05          -2         -13         -44         -11           0          -7
R=.6 B=-.10         -45         -10         -31          -1         -37         -61
R=.7 B= .00         -24          -2          -9         -90         -16          -3
R=.7 B=-.02          -5         -27          -3         -30           0         -23
R=.7 B=-.05          -7         -17          -2           0         -67          -8
R=.7 B=-.10          -3          -4         -14         -13         -25          -8
R=.8 B= .00         -21          -5         -21          -3         -30         -50
R=.8 B=-.02         -18          -2          -7           3         -15         -24
R=.8 B=-.05          -6         -24          -3         -37          -4         -27
R=.8 B=-.10         -36         -12          -1          -8         -77          -9
R=.9 B= .00          -2          -4         -26           0         -27          -2
R=.9 B=-.02         -24         -74          -5          -3          -6         -59
R=.9 B=-.05          -9          -1          -5         -39         -12         -21
R=.9 B=-.10          -4          -8         -74           0          -4          -6
```

$\mathrm{Var}\left( \hat{\rho}_{X} \right)$

```
            J= 6 N= 200 J= 6 N= 500 J= 6 N=2000 J=12 N= 200 J=12 N= 500 J=12 N=2000
R=.6 B= .00          19          13           1           4           0           2
R=.6 B=-.02           5           3           2           5           2           4
R=.6 B=-.05           1          19          42           1           3           0
R=.6 B=-.10          16           6           0           2           6           4
R=.7 B= .00           4           1          35           4           2           1
R=.7 B=-.02           0          17           4           1           4           6
R=.7 B=-.05           7           5           0          27           4           1
R=.7 B=-.10           1           1          22           2           1           1
R=.8 B= .00           9           8          18           0          11           5
R=.8 B=-.02           7           1           0          37           3           2
R=.8 B=-.05           1           2          14           2           0          13
R=.8 B=-.10           7           7           1           0           9           2
R=.9 B= .00           2           1           2          11           2           0
R=.9 B=-.02          15           1           9           0           0          10
R=.9 B=-.05           4           2           5           5           4           2
R=.9 B=-.10           2          11           1          15           1           0
```

$1/8\sigma_{\epsilon}^{3}\mathrm{Var}\left( \hat{\sigma}_{\epsilon}^{2} \right)$

```
            J= 6 N= 200 J= 6 N= 500 J= 6 N=2000 J=12 N= 200 J=12 N= 500 J=12 N=2000
R=.6 B= .00           2           7           2           2           0           1
R=.6 B=-.02           1           3           0           1           3           0
R=.6 B=-.05           0           3          26           1           2           0
R=.6 B=-.10           3           1           1           1           2          10
R=.7 B= .00           1           0          17           0           7           0
R=.7 B=-.02           0           5           2           1           3           2
R=.7 B=-.05           1           3           1          15           0           0
R=.7 B=-.10           0           1          11           0           1           0
R=.8 B= .00           4           1          11           0           0           2
R=.8 B=-.02           1           0           0          28           1           4
R=.8 B=-.05           0           6           7           0           2           2
R=.8 B=-.10           6           2           1           0           1           1
R=.9 B= .00           0           1           3           6           0           0
R=.9 B=-.02           3           2           5           0           0           2
R=.9 B=-.05           2           0           3          17           0           1
R=.9 B=-.10           0           6           2          11           0           1
```

### 3.2 Graphic Display of the Relationship Between Bias and Bias Approximation

Figure 1 in the paper shows the relationship between bias and the bias approximation for the SEM estimator. The figures show this relation for all five estimators of interest:

$\hat{\tau}\left( \theta=-2 \right)$ (top) and $\hat{\tau}(\theta=0$) (bottom)

$\hat{\tau}(\theta=2)$

$\hat{\sigma}_{E}$ (equals Figure 1 in the paper)

$\hat{\sigma}_{\epsilon}$

### 3.3 RMSE of the Standard errors

Table 4 in the paper provides the summary statistics of the distributions of the RMSEs of the proposed standard errors across the 96 study conditions. The tables below provide the RMSE (multiplied by 10,000 to facilitate readability for:

$\hat{\tau}(\theta=-2)$

```
            J= 6 N= 200 J= 6 N= 500 J= 6 N=2000 J=12 N= 200 J=12 N= 500 J=12 N=2000
R=.6 B= .00      0.0123      0.0137      0.0016      0.0047      0.0011      0.0042
R=.6 B=-.02      0.0018      0.0062      0.0022      0.0066      0.0035      0.0066
R=.6 B=-.05      0.0009      0.0142      0.0196      0.0024      0.0011      0.0009
R=.6 B=-.10      0.0217      0.0022      0.0025      0.0028      0.0036      0.0149
R=.7 B= .00      0.0042      0.0013      0.0429      0.0107      0.0011      0.0009
R=.7 B=-.02      0.0014      0.0203      0.0026      0.0051      0.0034      0.0083
R=.7 B=-.05      0.0048      0.0055      0.0004      0.0105      0.0087      0.0017
R=.7 B=-.10      0.0005      0.0017      0.0263      0.0026      0.0015      0.0016
R=.8 B= .00      0.0124      0.0057      0.0088      0.0006      0.0092      0.0135
R=.8 B=-.02      0.0087      0.0006      0.0006      0.0329      0.0014      0.0062
R=.8 B=-.05      0.0010      0.0039      0.0169      0.0044      0.0003      0.0046
R=.8 B=-.10      0.0151      0.0086      0.0007      0.0013      0.0158      0.0034
R=.9 B= .00      0.0012      0.0014      0.0057      0.0044      0.0035      0.0004
R=.9 B=-.02      0.0056      0.0060      0.0106      0.0007      0.0004      0.0102
R=.9 B=-.05      0.0050      0.0016      0.0021      0.0027      0.0037      0.0055
R=.9 B=-.10      0.0023      0.0064      0.0126      0.0141      0.0004      0.0016
```

$\hat{\tau}(\theta=0)$

```
            J= 6 N= 200 J= 6 N= 500 J= 6 N=2000 J=12 N= 200 J=12 N= 500 J=12 N=2000
R=.6 B= .00      0.0037      0.0078      0.0007      0.0025      0.0004      0.0013
R=.6 B=-.02      0.0032      0.0009      0.0004      0.0026      0.0044      0.0016
R=.6 B=-.05      0.0002      0.0045      0.0332      0.0011      0.0004      0.0004
R=.6 B=-.10      0.0045      0.0029      0.0010      0.0004      0.0059      0.0039
R=.7 B= .00      0.0036      0.0005      0.0239      0.0032      0.0047      0.0003
R=.7 B=-.02      0.0003      0.0039      0.0013      0.0026      0.0008      0.0024
R=.7 B=-.05      0.0015      0.0032      0.0002      0.0044      0.0036      0.0005
R=.7 B=-.10      0.0008      0.0002      0.0032      0.0010      0.0017      0.0004
R=.8 B= .00      0.0047      0.0018      0.0148      0.0003      0.0031      0.0034
R=.8 B=-.02      0.0018      0.0008      0.0002      0.0077      0.0024      0.0017
R=.8 B=-.05      0.0009      0.0018      0.0092      0.0013      0.0011      0.0079
R=.8 B=-.10      0.0021      0.0016      0.0003      0.0006      0.0039      0.0009
R=.9 B= .00      0.0004      0.0008      0.0026      0.0018      0.0015      0.0001
R=.9 B=-.02      0.0072      0.0023      0.0013      0.0003      0.0004      0.0087
R=.9 B=-.05      0.0019      0.0004      0.0034      0.0117      0.0012      0.0013
R=.9 B=-.10      0.0004      0.0032      0.0064      0.0033      0.0006      0.0005
```

$\hat{\tau}(\theta=2)$

```
            J= 6 N= 200 J= 6 N= 500 J= 6 N=2000 J=12 N= 200 J=12 N= 500 J=12 N=2000
R=.6 B= .00      0.0120      0.0283      0.0016      0.0004      0.0011      0.0032
R=.6 B=-.02      0.0067      0.0054      0.0022      0.0065      0.0120      0.0068
R=.6 B=-.05      0.0005      0.0095      0.0831      0.0010      0.0019      0.0014
R=.6 B=-.10      0.0211      0.0068      0.0024      0.0027      0.0123      0.0067
R=.7 B= .00      0.0099      0.0005      0.0038      0.0109      0.0081      0.0009
R=.7 B=-.02      0.0009      0.0219      0.0043      0.0010      0.0051      0.0054
R=.7 B=-.05      0.0047      0.0115      0.0004      0.0178      0.0126      0.0013
R=.7 B=-.10      0.0017      0.0014      0.0262      0.0026      0.0048      0.0017
R=.8 B= .00      0.0044      0.0040      0.0387      0.0003      0.0092      0.0089
R=.8 B=-.02      0.0087      0.0020      0.0006      0.0476      0.0049      0.0027
R=.8 B=-.05      0.0025      0.0039      0.0014      0.0044      0.0020      0.0167
R=.8 B=-.10      0.0128      0.0098      0.0012      0.0002      0.0158      0.0021
R=.9 B= .00      0.0012      0.0030      0.0025      0.0075      0.0052      0.0003
R=.9 B=-.02      0.0170      0.0060      0.0107      0.0007      0.0012      0.0240
R=.9 B=-.05      0.0018      0.0010      0.0088      0.0202      0.0036      0.0038
R=.9 B=-.10      0.0022      0.0107      0.0025      0.0212      0.0012      0.0007
```

$\hat{\sigma}_{E}$

```
            J= 6 N= 200 J= 6 N= 500 J= 6 N=2000 J=12 N= 200 J=12 N= 500 J=12 N=2000
R=.6 B= .00      0.0012      0.0011      0.0004      0.0002      0.0001      0.0010
R=.6 B=-.02      0.0004      0.0005      0.0001      0.0011      0.0014      0.0006
R=.6 B=-.05      0.0001      0.0008      0.0017      0.0005      0.0001      0.0001
R=.6 B=-.10      0.0013      0.0003      0.0005      0.0001      0.0011      0.0013
R=.7 B= .00      0.0003      0.0002      0.0013      0.0014      0.0006      0.0001
R=.7 B=-.02      0.0001      0.0007      0.0006      0.0006      0.0002      0.0012
R=.7 B=-.05      0.0005      0.0005      0.0001      0.0009      0.0009      0.0004
R=.7 B=-.10      0.0002      0.0001      0.0010      0.0005      0.0005      0.0001
R=.8 B= .00      0.0013      0.0003      0.0007      0.0002      0.0012      0.0010
R=.8 B=-.02      0.0005      0.0001      0.0002      0.0014      0.0006      0.0005
R=.8 B=-.05      0.0001      0.0011      0.0006      0.0006      0.0003      0.0011
R=.8 B=-.10      0.0011      0.0003      0.0001      0.0001      0.0014      0.0005
R=.9 B= .00      0.0001      0.0001      0.0013      0.0005      0.0004      0.0001
R=.9 B=-.02      0.0009      0.0014      0.0004      0.0001      0.0002      0.0008
R=.9 B=-.05      0.0005      0.0001      0.0002      0.0015      0.0005      0.0004
R=.9 B=-.10      0.0001      0.0013      0.0014      0.0006      0.0001      0.0001
```

$\hat{\sigma}_{\epsilon}$

```
            J= 6 N= 200 J= 6 N= 500 J= 6 N=2000 J=12 N= 200 J=12 N= 500 J=12 N=2000
R=.6 B= .00      0.0065      0.0033      0.0007      0.0005      0.0003      0.0015
R=.6 B=-.02      0.0009      0.0007      0.0007      0.0028      0.0017      0.0022
R=.6 B=-.05      0.0002      0.0053      0.0072      0.0007      0.0005      0.0001
R=.6 B=-.10      0.0079      0.0015      0.0009      0.0005      0.0014      0.0016
R=.7 B= .00      0.0016      0.0002      0.0054      0.0026      0.0006      0.0003
R=.7 B=-.02      0.0002      0.0066      0.0009      0.0006      0.0007      0.0016
R=.7 B=-.05      0.0025      0.0013      0.0002      0.0052      0.0014      0.0006
R=.7 B=-.10      0.0002      0.0002      0.0055      0.0011      0.0007      0.0005
R=.8 B= .00      0.0018      0.0021      0.0032      0.0002      0.0026      0.0016
R=.8 B=-.02      0.0032      0.0004      0.0002      0.0073      0.0006      0.0007
R=.8 B=-.05      0.0004      0.0018      0.0020      0.0012      0.0002      0.0024
R=.8 B=-.10      0.0018      0.0028      0.0002      0.0002      0.0051      0.0007
R=.9 B= .00      0.0006      0.0003      0.0016      0.0022      0.0006      0.0002
R=.9 B=-.02      0.0036      0.0022      0.0022      0.0003      0.0002      0.0039
R=.9 B=-.05      0.0007      0.0005      0.0007      0.0014      0.0011      0.0006
R=.9 B=-.10      0.0008      0.0020      0.0016      0.0031      0.0001      0.0002
```

### 3.4 Coverage Rate of the Standard Errors

Table 5 in the paper provides the summary statistics of the distributions of the Coverage Rates of the proposed standard errors across the 96 study conditions. The tables below provide the coverage rates for:

$\hat{\tau}(\theta=-2)$

```
            J= 6 N= 200 J= 6 N= 500 J= 6 N=2000 J=12 N= 200 J=12 N= 500 J=12 N=2000
R=.6 B= .00      0.9372      0.9370      0.9476      0.9472      0.9480      0.9416
R=.6 B=-.02      0.9496      0.9518      0.9488      0.9390      0.9410      0.9446
R=.6 B=-.05      0.9498      0.9406      0.9146      0.9452      0.9510      0.9542
R=.6 B=-.10      0.9342      0.9462      0.9448      0.9504      0.9446      0.9298
R=.7 B= .00      0.9476      0.9530      0.9386      0.9468      0.9496      0.9524
R=.7 B=-.02      0.9504      0.9386      0.9506      0.9448      0.9506      0.9428
R=.7 B=-.05      0.9486      0.9480      0.9522      0.9416      0.9432      0.9480
R=.7 B=-.10      0.9512      0.9466      0.9382      0.9492      0.9450      0.9496
R=.8 B= .00      0.9386      0.9424      0.9270      0.9470      0.9422      0.9445
R=.8 B=-.02      0.9480      0.9494      0.9524      0.9309      0.9534      0.9482
R=.8 B=-.05      0.9532      0.9502      0.9462      0.9462      0.9534      0.9432
R=.8 B=-.10      0.9432      0.9430      0.9522      0.9480      0.9366      0.9504
R=.9 B= .00      0.9534      0.9476      0.9428      0.9472      0.9454      0.9498
R=.9 B=-.02      0.9424      0.9442      0.9518      0.9510      0.9488      0.9408
R=.9 B=-.05      0.9448      0.9552      0.9436      0.9538      0.9472      0.9494
R=.9 B=-.10      0.9514      0.9488      0.9394      0.9368      0.9508      0.9534
```

$\hat{\tau}(\theta=0)$

```
            J= 6 N= 200 J= 6 N= 500 J= 6 N=2000 J=12 N= 200 J=12 N= 500 J=12 N=2000
R=.6 B= .00      0.9572      0.9408      0.9522      0.9464      0.9532      0.9542
R=.6 B=-.02      0.9446      0.9530      0.9530      0.9588      0.9462      0.9524
R=.6 B=-.05      0.9528      0.9504      0.9078      0.9476      0.9492      0.9496
R=.6 B=-.10      0.9560      0.9408      0.9470      0.9570      0.9420      0.9436
R=.7 B= .00      0.9474      0.9552      0.9284      0.9576      0.9382      0.9486
R=.7 B=-.02      0.9536      0.9550      0.9518      0.9494      0.9512      0.9560
R=.7 B=-.05      0.9496      0.9492      0.9520      0.9546      0.9512      0.9544
R=.7 B=-.10      0.9498      0.9458      0.9514      0.9548      0.9484      0.9552
R=.8 B= .00      0.9466      0.9486      0.9278      0.9546      0.9570      0.9494
R=.8 B=-.02      0.9528      0.9560      0.9502      0.9432      0.9422      0.9542
R=.8 B=-.05      0.9478      0.9614      0.9438      0.9488      0.9516      0.9418
R=.8 B=-.10      0.9504      0.9480      0.9478      0.9512      0.9580      0.9550
R=.9 B= .00      0.9516      0.9478      0.9472      0.9490      0.9522      0.9564
R=.9 B=-.02      0.9440      0.9604      0.9496      0.9520      0.9462      0.9418
R=.9 B=-.05      0.9478      0.9500      0.9462      0.9298      0.9528      0.9502
R=.9 B=-.10      0.9532      0.9528      0.9404      0.9460      0.9530      0.9572
```

$\hat{\tau}(\theta=2)$

```
            J= 6 N= 200 J= 6 N= 500 J= 6 N=2000 J=12 N= 200 J=12 N= 500 J=12 N=2000
R=.6 B= .00      0.9432      0.9353      0.9498      0.9502      0.9496      0.9486
R=.6 B=-.02      0.9472      0.9416      0.9526      0.9422      0.9360      0.9386
R=.6 B=-.05      0.9496      0.9442      0.9366      0.9420      0.9584      0.9480
R=.6 B=-.10      0.9418      0.9430      0.9474      0.9508      0.9370      0.9330
R=.7 B= .00      0.9464      0.9454      0.9516      0.9392      0.9386      0.9536
R=.7 B=-.02      0.9506      0.9454      0.9440      0.9504      0.9500      0.9372
R=.7 B=-.05      0.9478      0.9483      0.9458      0.9452      0.9404      0.9498
R=.7 B=-.10      0.9518      0.9472      0.9353      0.9492      0.9478      0.9500
R=.8 B= .00      0.9410      0.9446      0.9324      0.9466      0.9408      0.9408
R=.8 B=-.02      0.9476      0.9542      0.9506      0.9419      0.9462      0.9458
R=.8 B=-.05      0.9528      0.9466      0.9470      0.9474      0.9524      0.9444
R=.8 B=-.10      0.9450      0.9370      0.9436      0.9514      0.9438      0.9444
R=.9 B= .00      0.9506      0.9496      0.9476      0.9458      0.9442      0.9510
R=.9 B=-.02      0.9388      0.9454      0.9468      0.9502      0.9466      0.9420
R=.9 B=-.05      0.9426      0.9452      0.9442      0.9294      0.9488      0.9402
R=.9 B=-.10      0.9526      0.9472      0.9472      0.9445      0.9534      0.9510
```

$\hat{\sigma}_{E}$

```
            J= 6 N= 200 J= 6 N= 500 J= 6 N=2000 J=12 N= 200 J=12 N= 500 J=12 N=2000
R=.6 B= .00      0.9500      0.9498      0.9484      0.9532      0.9508      0.9360
R=.6 B=-.02      0.9516      0.9508      0.9504      0.9472      0.9478      0.9452
R=.6 B=-.05      0.9464      0.9524      0.9434      0.9476      0.9512      0.9490
R=.6 B=-.10      0.9524      0.9522      0.9488      0.9508      0.9502      0.9386
R=.7 B= .00      0.9496      0.9472      0.9502      0.9546      0.9508      0.9502
R=.7 B=-.02      0.9518      0.9520      0.9470      0.9524      0.9466      0.9454
R=.7 B=-.05      0.9528      0.9494      0.9542      0.9478      0.9492      0.9408
R=.7 B=-.10      0.9536      0.9466      0.9514      0.9546      0.9486      0.9490
R=.8 B= .00      0.9486      0.9470      0.9514      0.9460      0.9460      0.9444
R=.8 B=-.02      0.9476      0.9486      0.9526      0.9470      0.9536      0.9488
R=.8 B=-.05      0.9484      0.9514      0.9444      0.9522      0.9448      0.9436
R=.8 B=-.10      0.9454      0.9446      0.9486      0.9506      0.9512      0.9468
R=.9 B= .00      0.9524      0.9548      0.9498      0.9434      0.9474      0.9528
R=.9 B=-.02      0.9480      0.9532      0.9440      0.9480      0.9472      0.9490
R=.9 B=-.05      0.9468      0.9518      0.9528      0.9446      0.9492      0.9494
R=.9 B=-.10      0.9528      0.9410      0.9446      0.9484      0.9542      0.9478
```

$\hat{\sigma}_{\epsilon}$

```
            J= 6 N= 200 J= 6 N= 500 J= 6 N=2000 J=12 N= 200 J=12 N= 500 J=12 N=2000
R=.6 B= .00      0.8896      0.9390      0.9482      0.9458      0.9298      0.9366
R=.6 B=-.02      0.9478      0.9472      0.9516      0.9298      0.9438      0.9230
R=.6 B=-.05      0.9534      0.9252      0.9192      0.9482      0.9546      0.9438
R=.6 B=-.10      0.9142      0.9420      0.9480      0.9494      0.9506      0.9360
R=.7 B= .00      0.9432      0.9498      0.9404      0.9652      0.9492      0.9522
R=.7 B=-.02      0.9488      0.9358      0.9430      0.9512      0.9516      0.9486
R=.7 B=-.05      0.9250      0.9472      0.9530      0.9450      0.9524      0.9424
R=.7 B=-.10      0.9500      0.9476      0.9348      0.9508      0.9452      0.9458
R=.8 B= .00      0.9450      0.9408      0.9328      0.9462      0.9894      0.9354
R=.8 B=-.02      0.9402      0.9560      0.9512      0.9378      0.9572      0.9468
R=.8 B=-.05      0.9530      0.9504      0.9480      0.9250      0.9458      0.9542
R=.8 B=-.10      0.9458      0.9356      0.9494      0.9518      0.8956      0.9464
R=.9 B= .00      0.9482      0.9534      0.9490      0.9486      0.9536      0.9476
R=.9 B=-.02      0.9454      0.9534      0.9490      0.9472      0.9478      0.9414
R=.9 B=-.05      0.9468      0.9508      0.9486      0.9462      0.9886      0.9494
R=.9 B=-.10      0.9536      0.9464      0.9410      0.9422      0.9532      0.9504
```

### 3.5 Graphic Display of the Relationship Between Estimated and Empirical Standard Errors

## The figures below show the relationship between the estimated standard error using the proposed estimators under a multinomial sampling scheme and the empirical standard errors for the five parameter estimators of interest.

$\hat{\tau}(\theta=-2)$

$\hat{\tau}(\theta=0$)

$\hat{\tau}(\theta=2)$

$\hat{\sigma}_{E}$ (equals Figure 1 in the paper)

$\hat{\sigma}_{\epsilon}$

## 4. Accuracy in the Classical Method and Kelley’s Method

## Defined by the RMSE for the parameter estimators of interest, Table 6 in the paper provides the best-case and worst-case scenarios for small and large samples for Kelley’s method and the classical method. The tables below provide all 96 scenarios for each of the five parameter estimators of interest:

$\hat{\tau}(\theta=-2)$

```
            J= 6 N= 200 J= 6 N= 500 J= 6 N=2000 J=12 N= 200 J=12 N= 500 J=12 N=2000
R=.6 B= .00      0.9683      1.0514      0.3001      1.1241      1.0382      0.6414
R=.6 B=-.02      0.5434      0.9384      1.3449      0.5821      0.5277      1.3399
R=.6 B=-.05      0.7567      0.9826      0.5814      0.4630      0.7194      0.9169
R=.6 B=-.10      1.5307      0.5636      0.5862      1.1103      0.4119      1.0104
R=.7 B= .00      1.0049      0.9287      1.1491      1.0419      0.1196      0.8049
R=.7 B=-.02      1.0907      1.3547      0.8008      0.8227      0.9980      0.7591
R=.7 B=-.05      0.9653      1.0485      0.2997      0.7241      0.9198      0.6405
R=.7 B=-.10      0.5429      0.9360      1.1253      0.5806      0.5270      1.3378
R=.8 B= .00      0.9330      0.9785      0.5768      0.4624      0.8082      1.0960
R=.8 B=-.02      1.5247      0.5629      0.5855      1.0158      0.4111      1.0070
R=.8 B=-.05      1.0037      0.3010      1.1329      1.0395      0.1192      0.5443
R=.8 B=-.10      0.9429      1.3482      0.8001      0.8211      1.3439      0.7575
R=.9 B= .00      0.9638      1.0469      0.4643      0.7210      0.9179      0.6401
R=.9 B=-.02      0.5649      0.5875      1.1155      0.5799      0.5267      1.0073
R=.9 B=-.05      0.9302      0.9764      0.5741      0.1204      0.8060      1.0925
R=.9 B=-.10      1.5215      0.8021      0.8257      1.0045      0.4107      1.0051
```

$\hat{\tau}(\theta=0)$

```
            J= 6 N= 200 J= 6 N= 500 J= 6 N=2000 J=12 N= 200 J=12 N= 500 J=12 N=2000
R=.6 B= .00      0.0985      0.1057      0.0192      0.1658      0.0295      0.0452
R=.6 B=-.02      0.1115      0.1242      0.0590      0.0535      0.1373      0.0857
R=.6 B=-.05      0.1456      0.1168      0.1413      0.0936      0.0886      0.0551
R=.6 B=-.10      0.1765      0.1126      0.0320      0.0653      0.1349      0.1122
R=.7 B= .00      0.0934      0.1560      0.2902      0.0922      0.1086      0.0242
R=.7 B=-.02      0.0622      0.1729      0.0377      0.1500      0.1572      0.1578
R=.7 B=-.05      0.0627      0.0706      0.0096      0.1209      0.0920      0.0304
R=.7 B=-.10      0.1091      0.1042      0.1946      0.0338      0.1346      0.0429
R=.8 B= .00      0.1797      0.0743      0.1210      0.0904      0.0758      0.1243
R=.8 B=-.02      0.1129      0.1091      0.0160      0.2460      0.1325      0.0756
R=.8 B=-.05      0.0791      0.0303      0.2175      0.0588      0.1081      0.1162
R=.8 B=-.10      0.1546      0.1116      0.0190      0.1412      0.1343      0.1498
R=.9 B= .00      0.0314      0.0416      0.0997      0.1007      0.0698      0.0189
R=.9 B=-.02      0.1191      0.0504      0.1263      0.0170      0.1332      0.1163
R=.9 B=-.05      0.1645      0.0374      0.1074      0.1095      0.0483      0.0883
R=.9 B=-.10      0.0565      0.0591      0.1658      0.1943      0.1313      0.0442
```

$\hat{\tau}(\theta=2)$

```
            J= 6 N= 200 J= 6 N= 500 J= 6 N=2000 J=12 N= 200 J=12 N= 500 J=12 N=2000
R=.6 B= .00      0.9683      1.2930      0.3001      0.3831      1.0382      0.5455
R=.6 B=-.02      0.9860      1.0277      1.4062      0.5821      0.8914      1.3399
R=.6 B=-.05      0.5197      0.8901      1.3683      0.2905      1.1873      1.0650
R=.6 B=-.10      1.5307      1.1148      0.5862      1.3729      0.8090      0.7456
R=.7 B= .00      1.4548      0.5981      0.4511      1.0419      0.6369      0.8049
R=.7 B=-.02      0.9561      1.4155      0.7747      0.3992      1.3028      0.5233
R=.7 B=-.05      0.9653      1.2906      0.2997      1.1901      1.0675      0.5445
R=.7 B=-.10      0.9858      1.0255      1.3851      0.5806      0.8910      1.3378
R=.8 B= .00      0.6047      0.8855      1.3663      0.2895      0.8082      0.9621
R=.8 B=-.02      1.5247      1.1145      0.5855      1.3164      0.8086      0.7409
R=.8 B=-.05      1.4539      0.3010      0.4081      1.0395      0.6368      0.9866
R=.8 B=-.10      1.0318      1.4094      0.7740      0.3960      1.3439      0.5209
R=.9 B= .00      0.9638      1.2893      0.2925      1.1882      1.0658      0.5439
R=.9 B=-.02      1.1155      0.5875      1.3771      0.5799      0.8908      1.4564
R=.9 B=-.05      0.6003      0.8832      1.3652      0.6371      0.8060      0.9581
R=.9 B=-.10      1.5215      0.7760      0.4054      1.3078      0.8084      0.7384
```

$\hat{\sigma}_{E}$

```
            J= 6 N= 200 J= 6 N= 500 J= 6 N=2000 J=12 N= 200 J=12 N= 500 J=12 N=2000
R=.6 B= .00      0.0199      0.0851      0.0203      0.0455      0.0074      0.2764
R=.6 B=-.02      0.0337      0.1981      0.0358      0.0265      0.2150      0.0118
R=.6 B=-.05      0.0941      0.0305      0.1297      0.0951      0.0939      0.0649
R=.6 B=-.10      0.0157      0.0739      0.0204      0.0662      0.0475      0.3181
R=.7 B= .00      0.0458      0.1643      0.0508      0.0237      0.1200      0.0071
R=.7 B=-.02      0.1333      0.0383      0.1136      0.1096      0.1040      0.0956
R=.7 B=-.05      0.0125      0.0839      0.0102      0.0953      0.0671      0.2764
R=.7 B=-.10      0.0316      0.1979      0.0684      0.0167      0.2145      0.0059
R=.8 B= .00      0.1652      0.0273      0.1287      0.0944      0.0227      0.1342
R=.8 B=-.02      0.0098      0.0732      0.0102      0.1063      0.0439      0.3176
R=.8 B=-.05      0.0448      0.0320      0.0473      0.0149      0.1189      0.0376
R=.8 B=-.10      0.1984      0.0367      0.1133      0.1088      0.0188      0.0946
R=.9 B= .00      0.0062      0.0834      0.0967      0.0944      0.0657      0.2763
R=.9 B=-.02      0.0752      0.0323      0.0670      0.0084      0.2143      0.0475
R=.9 B=-.05      0.1646      0.0256      0.1283      0.1222      0.0143      0.1336
R=.9 B=-.10      0.0049      0.1141      0.1113      0.1048      0.0419      0.3174
```

$\hat{\sigma}_{\epsilon}$

```
            J= 6 N= 200 J= 6 N= 500 J= 6 N=2000 J=12 N= 200 J=12 N= 500 J=12 N=2000
R=.6 B= .00      0.0152      0.0466      0.0160      0.0135      0.0014      0.2041
R=.6 B=-.02      0.0170      0.1117      0.0132      0.0121      0.1704      0.0083
R=.6 B=-.05      0.0579      0.0205      0.0648      0.0773      0.0158      0.0421
R=.6 B=-.10      0.0305      0.0326      0.0149      0.0178      0.0302      0.2357
R=.7 B= .00      0.0247      0.0919      0.0335      0.0056      0.0951      0.0006
R=.7 B=-.02      0.0821      0.0336      0.0425      0.0901      0.0206      0.0592
R=.7 B=-.05      0.0097      0.0401      0.0080      0.0340      0.0433      0.2040
R=.7 B=-.10      0.0148      0.1109      0.0406      0.0076      0.1698      0.0041
R=.8 B= .00      0.0933      0.0138      0.0549      0.0768      0.0042      0.0839
R=.8 B=-.02      0.0197      0.0311      0.0074      0.0496      0.0282      0.2348
R=.8 B=-.05      0.0215      0.0253      0.0224      0.0030      0.0940      0.0209
R=.8 B=-.10      0.1134      0.0223      0.0411      0.0893      0.0129      0.0583
R=.9 B= .00      0.0048      0.0362      0.0783      0.0236      0.0425      0.2039
R=.9 B=-.02      0.0354      0.0236      0.0277      0.0037      0.1694      0.0302
R=.9 B=-.05      0.0924      0.0085      0.0485      0.0973      0.0018      0.0827
R=.9 B=-.10      0.0098      0.0451      0.0914      0.0336      0.0271      0.2343
```

## References

Bergsma, W. P. (1997). Marginal models for categorical data. Tilburg, The Netherlands: Tilburg University Press. Retrieved from: <http://stats.lse.ac.uk/bergsma/pdf/bergsma_phdthesis.pdf>

Kritzer, H. M. (1977). Analyzing measures of association derived from contingency tables. *Sociological Methods and Research, 5,* 35-50. <https://doi.org/10.1177/004912417700500401>

McGraw, K. O., & Wong, S. P. (1996). Forming inferences about some intraclass correlation coefficients. *Psychological methods*, *1*(1), 30-36. https://doi.org/10.1037/1082-989X.1.1.30

Van der Ark, L. A. (2025). Standard errors for reliability coefficients. *Psychometrika, 90*(5), 1679-1704.  <https://doi.org/10.1017/psy.2025.10050>
